# The Immunoexpression and Prognostic Significance of Stem Cell Markers in Malignant Salivary Gland Tumors: A Systematic Review and Meta-Analysis

**DOI:** 10.3390/genes16010037

**Published:** 2024-12-29

**Authors:** Eleni-Marina Kalogirou, Athina Tosiou, Stavros Vrachnos, Vasileios L. Zogopoulos, Ioannis Michalopoulos, Theodora Tzanavari, Konstantinos I. Tosios

**Affiliations:** 1 Faculty of Health Sciences, Metropolitan College, 15125 Athens, Greece; ttzanavari@mitropolitiko.edu.gr; 2 UFR d’Odontologie, Université Paris Cité, 75006 Paris, France; tosiouathina@gmail.com; 3 Private Practice, 14563 Athens, Greece; vrast2000@gmail.com; 4 Centre of Systems Biology, Biomedical Research Foundation, Academy of Athens, 11527 Athens, Greece; vzogopoulos@bioacademy.gr (V.L.Z.); imichalop@bioacademy.gr (I.M.); 5 School of Dentistry, National and Kapodistrian University of Athens, 11527 Athens, Greece; ktosios@dent.uoa.gr

**Keywords:** salivary gland tumor, salivary gland carcinoma, salivary gland malignancy, mucoepidermoid carcinoma, adenoid cystic carcinoma, stem cell, marker, c-KIT, KIT, CD117, systematic review

## Abstract

**Background/Objectives:** Salivary gland carcinomas encompass a broad group of malignant lesions characterized by varied prognoses. Stem cells have been associated with the potential for self-renewal and differentiation to various subpopulations, resulting in histopathological variability and diverse biological behavior, features that characterize salivary gland carcinomas. This study aims to provide a thorough systematic review of immunohistochemical studies regarding the expression and prognostic significance of stem cell markers between different malignant salivary gland tumors (MSGTs). **Methods:** The English literature was searched via the databases MEDLINE/PubMed, EMBASE via OVID, Web of Science, Scopus, and CINHAL via EBSCO. The Joanna Briggs Institute Critical Appraisal Tool was used for risk of bias (RoB) assessment. Meta-analysis was conducted for markers evaluated in the same pair of diseases in at least two studies. **Results:** Fifty-four studies reported the expression of stem cell markers, e.g., c-KIT, CD44, CD133, CD24, ALDH1, BMI1, SOX2, OCT4, and NANOG, in various MSGTs. Low, moderate, and high RoB was observed in twenty-five, eleven, and eighteen studies, respectively. Meta-analysis revealed an outstanding discriminative ability of c-KIT for adenoid cystic carcinoma (AdCC) over polymorphous adenocarcinoma [P(LG)A] but did not confirm the prognostic significance of stem cell markers in MSGTs. **Conclusions:** This study indicated a possible link between stem cells and the histopathological heterogeneity and diverse biological behavior that characterize the MSGTs. c-KIT might be of diagnostic value in discriminating between AdCC and P(LG)A.

## 1. Introduction

Salivary gland tumors (SGTs) account for 3–10% of all head and neck tumors [1,2]. In the latest World Health Organization (WHO) Classification of Head and Neck Tumors, 15 benign SGTs (BSGTs), also described as adenomas, and 21 malignant SGTs (MSGTs), also referred to as salivary gland carcinomas or adenocarcinomas, are recognized [3]. This classification is primarily based on architectural patterns and cellular composition and predicts their varying biological behavior [3]. On clinical examination, SGTs present as asymptomatic or painful swellings, while imaging techniques, such as ultrasonography, computer tomography, or magnetic resonance imaging, provide valuable information regarding the dimensions and the predominant growth pattern of the tumors [4]. A recent multicentric study encompassing data from Europe, Africa, Asia, and South America analyzed 5739 cases of SGTs over a 14-year period [5]. BSGTs represented 65% of these, involved predominantly the parotid glands (70%), mostly in patients in their sixth decade of life, and showed a slight female predominance (female-to-male ratio: 1.2:1) [5]. Pleomorphic adenomas (PAs) and Warthin’s tumors (WTs) comprised more than 50% of the cases [5]. MSGTs were more commonly found in the minor salivary glands (47%) or the parotid glands (42%), in patients primarily between the sixth and eighth decades of life, with an almost equal gender distribution (female-to-male ratio: 1.1:1) [5].

MSGTs are rare tumors with an annual incidence for 2021 of 1.3 cases per 100,000 individuals [6]. The most common MSGT is mucoepidermoid carcinoma (MEC), which accounts for approximately 27% of MSGT cases [5]. MEC primarily involves the parotid glands and the minor salivary glands, particularly those of the palate, and less frequently affects the submandibular or sublingual glands. The average age at diagnosis is in the fifth decade of life, with no significant sex predilection [7,8]. Clinically, MEC often presents as an asymptomatic, slow-growing mass with a red-bluish hue and a soft and occasionally fluctuating, rubbery, or fixed consistency. In some cases, it may manifest with pain, discomfort, or surface ulceration [7,8]. Microscopically, MEC is characterized by the presence of mucous, epidermoid, and intermediate cells, with columnar, clear cell, apocrine, and oncocytoid changes. The tumor usually presents variably sized cystic spaces containing mucoid material but may also have a solid architectural pattern [7]. Based on various histological criteria, such as architectural pattern and cytological features, MECs are characterized as low-, intermediate-, or high-grade tumors [9], with the five-year survival rate for low-grade tumors being 98.8% and for high-grade tumors 67% [10].

The second most common MSGT is adenoid cystic carcinoma (AdCC), accounting for approximately 17% of MSGT cases [5]. AdCC predominantly arises in the minor salivary glands and typically affects patients in their sixth to seventh decades of life, with a female predilection [11]. Despite its early presentation as a slow-growing swelling, AdCC is associated with poor prognosis mostly due to its high propensity for local recurrence and distant metastases, usually to the lungs, skeleton, liver, and brain, with fatal outcomes [3,12,13]. Histologically, AdCC is composed of ductal and myoepithelial cells organized into three distinct architectural patterns: (1) the cribriform pattern, characterized by numerous pseudocysts and true cysts containing a basophilic extracellular matrix that give the tumor a “punched out” appearance; (2) the tubular pattern, showing ductal and tubular structures on a hyaline stroma; and (3) the solid pattern, consisting of compact sheets of cells in scant stroma [3,14]. A hallmark histopathological feature of AdCC is perineural invasion that is often associated with neurological symptoms. This feature is linked to poor prognosis, as the 10-year disease-free survival rate is less than 50% in the presence of perineural invasion [15].

Other less common MSGTs include polymorphous adenocarcinoma (formerly polymorphous low-grade adenocarcinoma, P(LG)A), carcinoma ex pleomorphic adenoma (CXPA), acinic cell carcinoma (ACC), and adenocarcinoma, not otherwise specified (NOS), which in the fifth edition of the WHO classification was included within the broader category of “salivary carcinoma, NOS and emerging entities” [3]. These subtypes account for 12%, 11%, 9%, and 7% of malignant SGTs, respectively [5].

Remarkable features of MSGTs, besides their variable histopathological appearances, are divergent molecular profiles and different biological behaviors. The concept of cancer stem cells (CSCs)—a subpopulation of tumor cells with properties reminiscent of embryonic stem cells, including resistance to apoptosis, self-renewal, and the ability to differentiate into multiple cell lineages—may provide a partial explanation for MSGTs’ heterogeneity and growth characteristics [16]. CSCs have been implicated in the pathogenesis of both hematological malignancies, such as leukemia, and solid tumors, including carcinomas of the breast, cervix, liver, lung, stomach, pancreas, prostate, colorectum, central nervous system, and head and neck [17]. Furthermore, CSCs have been linked to resistance to radiotherapy and chemotherapy [18], which are leading causes of MSGT recurrence following treatment [19].

Previous studies investigated the presence of stem cells in various MSGTs, though some findings have been ambiguous, conflicting, or contradictory [20]. Moreover, the expression of stem cell markers has been reported to exert a significant role in the biological behavior of MSGTs. For example, increased SOX2 expression was significantly correlated with the absence of myoepithelial differentiation among MSGTs [21]. CD24 and CD44 have been regarded as negative prognostic factors for MSGTs, associated with increased tumor size, positive lymph nodes, and advanced clinical stage [20,22]. The expression of OCT4/POU5F1 and NANOG was correlated with increased risk for adverse histopathological prognostic factors, i.e., desmoplasia and perineural invasion, in MEC [23], while BMI1 expression was an adverse prognostic factor in AdCC, associated with shorter overall and disease-free survival [24]. Notably, no comprehensive comparative analysis of stem cell marker expression across the spectrum of MSGTs has yet been undertaken. Identifying tumor cells with stem cell-like properties, as indicated by the expression of specific stem cell markers, and targeting them with novel, specialized therapies holds promise for improving therapeutic outcomes and patient survival [17].

The aim of the present study is to systematically summarize the evidence of pertinent literature on the immunoexpression and prognostic significance of stem cell immunohistochemical markers in MSGTs.

## 2. Materials and Methods

### 2.1. Research Questions and Study Protocol

The primary question of the study was “Are there any differences in the immunoexpression of stem cell markers between various MGSTs” and was formulated using the Population, Intervention, Comparator, and Outcome (PICO) framework, i.e., P = SGTs, Intervention = not applicable, Comparator = stem cell markers, Outcome-1 = differences in immunoexpression. The null hypothesis for this question is that there are no significant differences in the stem cell markers’ immunoexpression between different MGSTs.

A secondary question was also formulated utilizing the PICO framework as follows: “Is there any prognostic significance of the immunoexpression of the various stem cell markers in MGSTs?” (P = SGTs, Intervention = not applicable, Comparator = stem cell markers, Outcome-2 = prognostic significance of immunoexpression). The null hypothesis for the latter question is that there is no prognostic significance of the expression of the stem cell markers in MSGTs.

The study protocol was designed to adhere to the Preferred Reporting Items for Systematic Reviews and Meta-Analyses (PRISMA) guidelines [25] and was registered in the Prospective International Registration of Systematic Reviews database [26] (reference number CRD42024511543).

### 2.2. Search Strategy

A thorough literature search was carried out on November 1, 2023, using the databases MEDLINE/PubMed^®^, EMBASE via OVID, Web of Science, Scopus and CINAHL via EBSCOhost for original studies written in the English language that included in their title or abstract the following keywords: ((salivary OR “salivary gland”) AND (tumo* OR mass* OR neoplas* OR malignan* OR cancer* OR carcinoma* OR adenocarcinoma* OR “glandular carcinoma*” OR “glandular cancer*”)) AND (“stem cell*” OR pluripoten*) AND (immunohistochem* OR immunostain* OR stain* OR immunolab* OR immunocytochem* OR immunoexpress* OR immunofluorescence* OR antibod* OR protein* OR marker* OR biomarker*). Table S1 summarizes the search term algorithms that were used in each database. A manual search of the reference lists of retrieved articles was also performed to identify additional eligible studies. All the articles identified through the literature search were imported into a citation manager (EndNote^ΤΜ^ X8), and automatic removal of the duplicates was executed at the initial phase.

### 2.3. Eligibility Criteria and Study Selection

Screening of titles and abstracts of the non-duplicated articles was performed by three researchers (E.M.K., A.T., S.V.) independently to identify immunohistochemical studies of salivary gland carcinomas reporting the expression of stem cell markers and, if available, their prognostic significance. At this phase, (1) non-English studies, (2) non-original studies (i.e., case reports/short case studies/letters to the editor/correspondence/commentaries, narrative or systematic reviews, meeting abstracts), and (3) non-immunohistochemical studies, in vitro, or animal studies were rejected. If the suitability of the study could not be determined from the title/abstract, the full text was downloaded and evaluated. The same three researchers (E.M.K., A.T., S.V.) assessed the full texts independently for eligibility by applying the following inclusion criteria: (1) original studies reporting the immunoexpression of stem cell markers on fresh-frozen or formalin-fixed, paraffin-embedded (FFPE) human tissue samples of MSGTs; (2) comparing the immunoexpression of stem cell markers between at least two different MSGT categories (types), or between one MSGT group compared with a BSGT group or/and a normal salivary gland control group, or between different subgroups of one MGST category, e.g., histopathological subtypes of AdCC (cribriform, tubular, solid), or histopathological grading groups of MEC or AdCC (low-grade, intermediate-grade, high-grade), accompanied by statistical analysis or not; and (3) that were written in English and published in peer-reviewed scientific journals. Disagreements were resolved by asking for an opinion from a fourth author (K.I.T.). Eligible markers were regarded as the proteins known to exert a core role in stem cell phenotype induction and/or maintenance, as well as surface markers of ESCs or gene markers of cancer stem cells [27,28]. In studies analyzing the expression of multiple gene markers, the eligibility criteria were implemented for each marker individually. A list of studies excluded at the full-text evaluation phase and the reasons for exclusion are provided in Table S2.

### 2.4. Data Extraction and Qualitative Synthesis

Three researchers (E.M.K., A.T., S.V.) extracted information regarding (1) the general characteristics of included studies, (2) the characteristics of study population groups, (3) the details about the immunohistochemical method, and (4) the results of each study, into Microsoft Excel 365 spreadsheets (Microsoft Corporation, Redmond, WA, USA). Table S3, organized by the most recent study, provides data about the journal of publication; the number of patients and their demographics (gender, age); the study groups of MSGTs or BSGTs and normal controls, as well as their site (i.e., in major or minor salivary gland of head and neck); and, if available, the histopathological subtypes in case of AdCC, and the histopathological grades of MEC. Table S4, sorted by the protein name of each stem cell marker in alphabetical order, summarizes the details of the immunohistochemical experiment, i.e., the protein marker evaluated and the Human Genome Organization (HUGO) Gene Nomenclature gene symbol [29], the antibody characteristics (i.e., host, clonality, dilution catalog number, source company); the thickness (in μm) of tissue sections used for immunohistochemical or immunofluorescence staining; the positive and negative controls; the number of observers of the immunohistochemical sections; the method of staining evaluation, i.e., dichotomous (positive/negative), quantitative or semi-quantitative; and the applied scoring system, based on the intensity and/or extent of staining. Table S5, sorted by the protein name of the stem cell marker in alphabetical order, outlines the immunoexpression results, i.e., the ratio of positive cases/total cases; the pattern of immunoexpression in tissue (parenchyma, stroma), cellular (acinar cells, ductal cells, myoepithelial cells) and subcellular (nucleus, cytoplasm, membrane) level; the results of the comparison of stem cell markers’ expression between the study groups and the findings regarding the prognostic significance of this expression, i.e., any significant association between the expression of stem cell markers and prognostic factors, such as histopathological grade, clinical stage, extent of the tumor, presence of lymph node metastasis or distant metastasis, and overall or/and disease-free survival; and the methods of statistical analysis utilized.

### 2.5. Risk of Bias Assessment

The two authors most experienced in immunohistochemical studies (E.M.K., K.I.T.) independently assessed the risk of bias (RoB) in the eligible studies, and any cases with discrepancies were resolved through discussion until agreement was reached. The Critical Appraisal Tool, proposed by the Joanna Briggs Institute (JBI) [30], was employed to estimate the RoB of retrospective observational studies with relatively small cohorts and without focus on long-term follow-up data, as previously stated [31], and was appropriately adjusted based on the qualitative characteristics of the included studies of the present systematic review (Table S6). Each item in the JBI RoB tool was marked as “Yes” (low risk), “No” (high risk), “Unclear” (moderate/unclear risk), or “Not applicable” for each study. In studies with multiple stem cell markers, scoring was performed for each stem cell marker independently, and a final “Yes” was decided only if “Yes” applied to all markers, while a final “No” was scored if at least one marker was graded with “No”. Based on the final percentage of “Yes”, the RoB of each study was classified as high (≤49% “Yes”), moderate (50–69% “Yes”), or low (≥70% “Yes”).

### 2.6. Meta-Analysis

Quantitative analysis was performed by two researchers (V.L.Z, I.M.) to evaluate the discriminative ability of stem cell markers between various disease groups and, in case of available data, the ability of those markers to predict a worse prognosis. A marker was considered eligible for meta-analysis if it was tested in the same disease pair in at least two studies (or in the same study using two different antibodies for the same marker). For each marker and disease pair, a meta-analysis was conducted on the studies that passed the selection criteria, using Meta-DiSc Version 1.4 (Unit of the Clinical Biostatistics team of the Ramón y Cajal Hospital in Madrid, Spain) [32] as previously reported [31].

## 3. Results

### 3.1. Study Cohort

**Figure 1:**
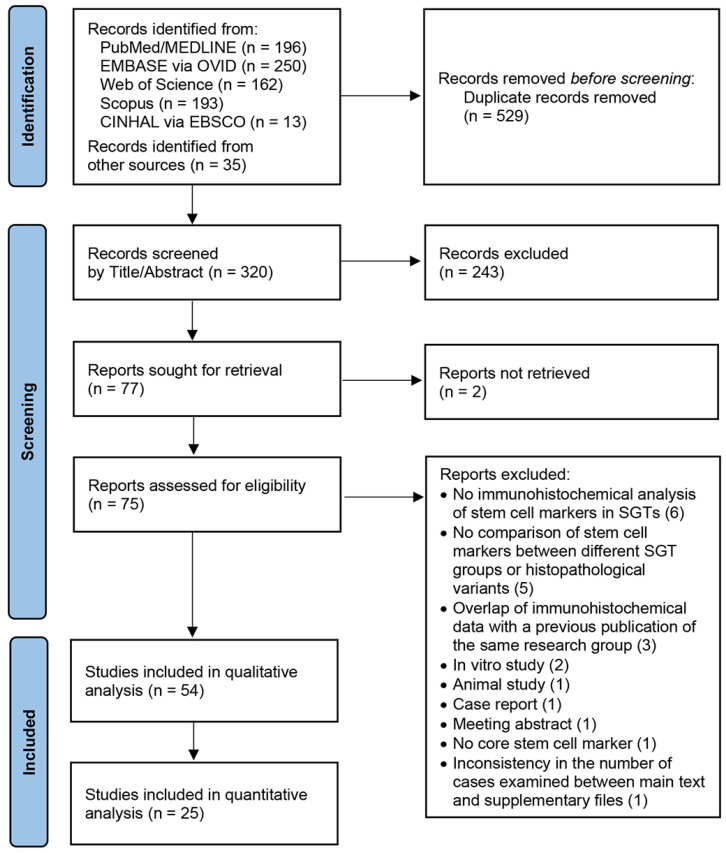
The PRISMA [25] flow diagram presenting the search strategy.

A total of 849 records were identified through the systematic search of the literature, i.e., 814 records via the five electronic databases and 35 records through manual search (Figure 1). First, 529 duplicates were removed. Then, the titles and abstracts of 320 records were screened, and 243 studies were rejected because of language other than English, study type other than original research, or content irrelevant to study aims. At the next search step, the full texts of 77 studies were evaluated for eligibility; two full texts could not be retrieved [33,34], and 21 studies did not meet the eligibility criteria and were excluded. Table S2 presents the reason for the exclusion of each study at this phase. Finally, 54 studies (Table 1) published in 43 scientific journals (Table S3) from 1998 to 2023 were considered eligible for qualitative synthesis, while 25 studies included data suitable for meta-analysis (Figure 1).

### 3.2. Characteristics of Study Population Groups

The immunoexpression of stem cell markers was evaluated in 19 different groups of MSGTs in 54 studies (Table S3). The two most encountered MSGTs were AdCC and MEC, which were included in 43 and 27 studies, respectively, followed by P(LG)A (17 studies), ACC (14 studies), CXPA (11 studies), and NOS (11 studies). Three types of comparisons were observed:

(1) Thirty-one studies comprised exclusively of patients with MSGTs. Nineteen out of 31 studies included only one MSGT category (type) and compared the immunoexpression of stem cell markers between different histopathological patterns of AdCC (13 studies); different histopathological grades of MEC (3 studies); different histopathological variants of MEC, i.e., cystic and solid (1 study); luminal and non-luminal cases of CXPA (1 study); or primary versus metastatic squamous cell carcinoma (SCC) of the SGs (1 study). In 5/19 studies, normal SGs derived from the same patients with the MSGTs were also used as control samples [43,44,62,71,74]. Six studies analyzed the immunoexpression of stem cell markers in two different MSGT categories, i.e., AdCC vs. P(LG)A (four studies) and MEC vs. AdCC (two studies), while eight studies included multiple (5 to 15) categories of MSGTs.

(2) Seventeen studies included both MSGTs (1 to 8 categories per study) and BSGTs (1 to 5 categories per study), and a control group of normal SGs or non-neoplastic SG lesions (e.g., sialadenitis group) was also added in 12/17 studies. Seven different BSGT categories were included in the 54 studies (Table 1). The most frequently encountered BSGT category was PA, which appeared in 15/17 studies. Two studies conducted by the same research group used the same patients with PA but compared them with different types of MSGTs [57,61].

(3) Six studies performed a comparative analysis of stem cell markers’ expression between one group of patients with MSGTs (MEC or AdCC) and another group of subjects with normal SGs.

The number of total patients per study ranged between 13 and 199 (Table S3). Information regarding gender and age of SGT cases was available in 48 and 47 studies, respectively, while the localization of SGTs was available in 46 studies and included major salivary glands, minor salivary glands, as well as salivary glands from other head and neck locations (Table S3). Of note, data about demographics and/or sites of the control group of normal salivary glands/non-neoplastic salivary gland lesions were missing in 11 and 10 studies, respectively, that provided relevant information for the SGT groups (Table S3). Thirty-one studies provided sufficient documentation on the histopathological criteria that guided the diagnosis of the included SGTs. Data on the histopathological grades of MEC or the histopathological patterns of AdCC were available in 16 and 26 studies, respectively.

### 3.3. Stem Cell Markers Comparators

The expression of fourteen different stem cell markers was investigated in the 54 studies included in the qualitative synthesis. Forty studies assessed one marker, and fourteen studies included two to five stem cell markers (Table 1). Four markers (ABCG2, CD10, CD166, and DCLK1) were “single”, i.e., each of them was included in one study, while ten markers (ALDH1, BMI1, CD133/PROM1, CD24, CD44, c-KIT/CD117, EZH2, NANOG, OCT4/POU5F1, and SOX2) were “repeated” and their expressions were evaluated in multiple studies. c-KIT expression was assessed in 21 studies, CD44 in nineteen studies, ALDH1 and SOX2 in eight studies each, BMI1 in seven studies, CD133 in six studies, and one of them [58] reported immunoexpression using two different anti-CD133 antibodies (Table S4). The positivity of CD24, NANOG, and OCT4 was investigated in three studies, and the expression of EZH2 was investigated in two studies (Table S4).

Immunohistochemistry on FFPE samples was performed in all studies except for the study by Adams et al. [54], which evaluated the immunoexpression of stem cell markers via immunofluorescence without specifying whether they used fresh-frozen or FFPE tissue samples. Table S4 summarizes the technical details of the immunohistochemical experiments, e.g., host species, clonality, dilution, and source company/catalog number. In most studies evaluating the expression of c-KIT, a rabbit polyclonal antibody was selected. In contrast, a mouse or rabbit monoclonal antibody against CD44 was always selected (Table S4). Five different antibodies derived from seven companies were used against c-KIT, while variation in the dilution of the same antibody was observed, e.g., A4502 was tested at 1:50 to 1:200 dilutions (Table S4). Similarly, anti-CD44 antibodies sourced from nine companies were tested at 1:50 to 1:400 dilutions (Table S4). Various positive controls were selected for the same marker, with gastrointestinal stromal tumor and tonsils being the most preferred for c-KIT and CD44, respectively. As negative controls, the omission of the primary antibody or the replacement of the primary antibody with non-immune serum, bovine serum albumin in phosphate-buffered saline, the same immunoglobulin class and subclass as the primary antibody, or isotype control were mostly reported (Table S4). The staining results were evaluated by one to three experienced observers, who applied dichotomous (positive/negative), semiquantitative (e.g., sum or product of extent and intensity scores), or quantitative (e.g., mean percentage of positive cells) scoring systems (Table S4). One study applied both a semiquantitative and a dichotomous method for the grading of staining in the tumor parenchyma and stroma, respectively [21], while two studies reported separate scoring systems for the extent and the intensity of staining, utilizing a quantitative and a semi-quantitative/qualitative method, respectively [50,81].

### 3.4. Stem Cell Markers’ Expression

The immunoexpression of all evaluated stem cell markers was confirmed in at least one MSGT category (type). Most studies documented the expression of stem cell markers in tumor parenchyma, while stromal expression was also reported in a few studies (Table S5).

#### 3.4.1. Mucoepidermoid Carcinoma

The expression of twelve stem cell markers was evaluated in MEC (Table S5). Most studies agreed on a patchy membranous CD44 expression, with variable intensity in epidermoid, intermediate, and mucous cells of MEC in 100% of evaluated cases, while focal cytoplasmic expression in epithelial cells and/or stromal expression was also described [21,41]. Nuclear expression of BMI1 and SOX2 and cytoplasmic or membranous staining of CD166 was seen in the tumor parenchyma, while mainly nuclear and occasionally cytoplasmic expression of OCT4 was observed in both parenchymal and stromal cells of MEC in most cases. Tumor cells of MEC were also found to express CD10. In contrast, limited membranous or cytoplasmic expression of c-KIT and weak expression of NANOG were shown to be expressed in less than 50% or 25% of MEC cases, respectively, while DCLK1 staining was not specified. Conflicting results regarding the immunostaining of ALDH1, CD24, and CD133 were observed. Some studies reported cytoplasmic ALDH1 expression in a high [39,54] or low [20] number of MEC cases, with stronger [39] or weaker [21] expression in mucous cells compared to epidermoid or intermediate cells; stromal expression was also described [21]. Membranous or cytoplasmic expression of CD24 was seen in the majority of MEC cases in one study [54] but only in a few cases in other studies [22,39]. Similarly, two studies reported CD133 expression in more than 84% [48] or 100% [38] of MEC cases, whereas another study utilizing two anti-CD133 antibodies derived from a different company reported staining in 0% or 13.3% of MEC cases [58].

#### 3.4.2. Adenoid Cystic Carcinoma

The immunostaining of thirteen stem cell markers was assessed in AdCC (Table S5). Twenty studies confirmed the positivity of c-KIT in most cases of AdCC. In almost all evaluated cases, c-KIT expression was observed in the cell membrane and/or the cytoplasm of the luminal (ductal) cells, in particular, in the inner cell layer of the cribriform and tubular patterns, and all luminal cells of the solid pattern, while myoepithelial cells were negative. Ductal and myoepithelial cells of most AdCC cases showed membranous and occasionally cytoplasmic expression of CD44, CD133, and ABCG2, and nuclear and occasionally cytoplasmic expression of OCT4. Nuclear SOX2, nuclear or cytoplasmic BMI1, and membranous or cytoplasmic CD24 or CD166 positivity were observed in the tumor cells of AdCC. Immunostaining results regarding DCLK1, EZH2, and NANOG in AdCC were not clear, while those regarding ALDH1 were contradictory. One study reported ALDH1 staining in both ductal and myoepithelial cells [21]; another highlighted expression in both the epithelium and/or stroma [62], while a third concluded that all AdCC cases were negative [39].

#### 3.4.3. Polymorphous Adenocarcinoma

The expression of eight stem cell markers was explored in P(LG)A (Table S5). Membranous or cytoplasmic CD44 staining was observed in ductal and myoepithelial cells, while tumor cells of most studied P(LG)A cases also expressed ALDH1 and CD166. CD24 and c-KIT showed limited membranous or cytoplasmic immunostaining in up to 50% of evaluated cases, while data on BMI1, NANOG, and SOX2 expression were not clearly presented.

#### 3.4.4. Acinic Cell Carcinoma

The positivity of ten stem cell markers was assessed in ACC (Table S5). CD44 showed membranous expression in most ACC cases. In contrast, c-KIT was expressed in the cell membrane or cytoplasm in less than 50% of evaluated cases, and ALDH1, CD24, and CD166 were positive in a limited number of cases. CD133 was reported to stain positive 10/11 and 1/11 cases of ACC in one study employing two anti-CD133 antibodies, while data on BMI1, DCLK1, NANOG, and SOX2 expression in ACC were not comprehensive.

#### 3.4.5. Carcinoma Ex Pleomorphic Adenoma

The expression of nine stem cell markers was evaluated in CXPA (Table S5). SOX2 nuclear expression was observed in intraductal, extraductal intracapsular, and extracapsular areas of almost all CXPA cases, while cytoplasmic expression of ALDH1, membranous or cytoplasmic expression of CD24 and CD166, and membranous expression of CD44 was confirmed in a small number of cases. On the other hand, c-KIT showed membranous and/or cytoplasmic expression in less than 50% of studied cases.

#### 3.4.6. Adenocarcinoma, Not Otherwise Specified

The immunostaining of eight stem cell markers was investigated in NOS (Table S5). Membranous or cytoplasmic expression of CD44 and c-KIT was observed in all and limited cases, respectively. Absence of CD24 expression was documented in a small number of NOS cases, while clearly specified data on the expression of ALDH1, BMI1, DCLK1, NANOG, and SOX2 were not available.

#### 3.4.7. Other MSGTs

A limited body of evidence regarding the immunoexpression of stem cell markers in the remaining MSGTs was available, derived from a small number of studies (Table S5). A few studied cases of salivary duct carcinoma (SDC) were positive for CD24 and CD44, while almost all cases were negative for c-KIT. Myoepithelial carcinoma (MYOC) was positive for BMI1 while showing scarce positivity for c-KIT. In contrast, the epithelial–myoepithelial carcinoma (EMC) was c-KIT-positive in most of the evaluated cases. Expression of BMI1, CD44, and SOX2 was confirmed in all cases of SCC, while rare positivity for ALDH1 and c-KIT was observed. CD44 expression was observed in a few cases of basal cell adenocarcinoma (BCAC) or clear cell carcinoma. c-KIT expression was confirmed in lymphoepithelial carcinoma (LEP) but in a few cases of BCAC, oncocytic carcinoma, cystadenosarcoma, adenosquamous carcinoma, and undifferentiated carcinoma.

### 3.5. Prognostic Significance of Stem Cell Markers’ Expression

In 32/54 studies included in the qualitative synthesis, the correlation between the immunoexpression of eleven stem cell markers and prognostic variables was assessed (Table S5).

The prognostic significance of CD44 expression was evaluated in 13 studies; nine studies did not find any significant correlation; two studies considered CD44 expression to be a positive prognostic factor associated with low histological grade, absence of necrosis, and lower number of mitoses in MEC cases [37], or with early tumor stage, absence of distance metastasis, and survival in MEC and AdCC cases [21]; one study regarded CD44 as a negative prognostic factor for MSGTs (prognostic data not specified per histopathological subtype), significantly associated with higher tumor size, advanced tumor stage, and positive lymph nodes [20]; and another study concluded that CD44 is a negative prognostic factor, correlated with worst overall survival in MEC cases showing concurrent SOX2 expression, but not in MECs expressing only CD44 [48].

Nine studies explored the usefulness of c-KIT expression as a prognostic factor (Table S5). Five out of nine studies included AdCC cases, and only 2/9 studies revealed a significant correlation between c-KIT expression and negative prognostic factors, i.e., presence of perineural invasion, local regional recurrence, advanced tumor stage, distant metastasis [67], or decreased survival [66].

Eight studies assessed the prognostic significance of SOX2 positivity (Table S5). In one study with different categories (types) of MSGTs, SOX2 was regarded as a favorable prognostic variable, as high expression of this marker was associated with the absence of perineural invasion (prognostic data not specified per histopathological subtype) [20]. Another study found low SOX2 expression to be significantly correlated with high-grade MEC [38]. In contrast, SOX2 was considered a negative prognostic factor in four studies. SOX2 expression was significantly correlated with increased clinical stage [44] or greater tumor size, distant metastasis, and decreased overall and disease-free survival [56] in AdCC. Diffuse SOX2 expression was significantly correlated with high histological grade, mitoses, greater tumor size, recurrence, distant metastasis, and worst overall survival among patients with extracapsular CXPA [47]. In addition, low SOX2 expression was significantly associated with increased recurrence-free survival in SCC cases [40]. In two studies, significant association was not observed between SOX2 expression and prognostic variables [21,48].

Two studies reported ALDH1 expression as the worst prognostic factor for MSGTs (prognostic data not specified per histopathological type of MSGT), significantly associated with higher histopathological grade [20] or increased incidence of lymph node metastasis, advanced stage, increased recurrence, and higher number of deaths due to the disease [42]; however, significant prognostic correlation was not confirmed in four other studies including MEC, AdCC, or SCC cases (Table S5).

Two studies agreed that BMI1 expression is a negative prognostic variable in AdCC, correlated significantly with increased tumor size [24], higher tumor stage [24], presence of distant metastasis [24,53], and shorter overall and disease-free survival [24], while prognostic significance was not confirmed in three other studies including AdCC cases (Table S5). Moreover, low BMI-1 expression was significantly associated with better overall survival in one study with SCC cases [40].

One study reported CD133 expression to be an independent negative prognostic factor in AdCC, significantly associated with larger tumor size, local regional recurrence, distant metastasis, and reduced survival [52], whereas the prognostic significance of this marker was not confirmed in two other studies, including AdCC and/or MEC cases [38,48].

One study reported CD24 expression to be the worst prognostic variable for MSGTs (prognostic data not specified per histopathological type of MSGT), significantly correlated with higher tumor size, advanced tumor stage, and positive lymph nodes [22], while another study concluded against the prognostic significance of this marker [39].

The expression of OCT4 and NANOG was significantly correlated with adverse prognostic factors, i.e., desmoplasia and perineural invasion in MEC samples [23], while the prognostic significance of these markers was not confirmed in other studies [20,21,48].

High EZH2 expression was observed to be a negative prognostic factor, significantly associated with decreased survival of patients with AdCC [74]. High DCLK1 expression in major SGTs was a worse prognostic factor, associated with decreased overall and disease-free survival in patients with MSGTs of the major salivary glands [46].

### 3.6. RoB Assessment

**Figure 2:**
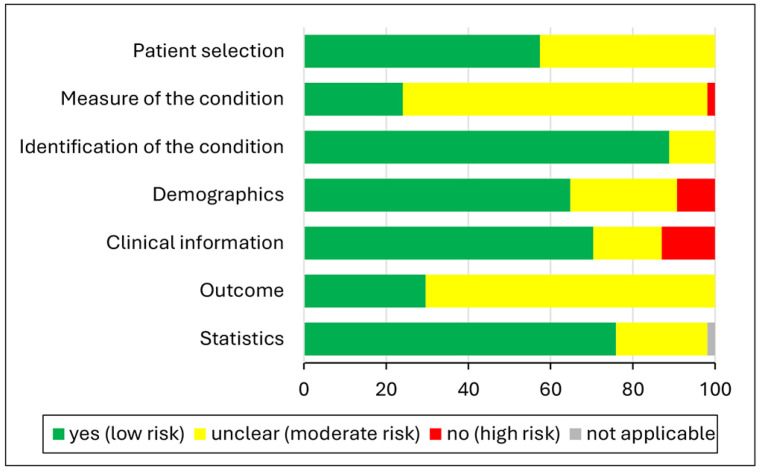
RoB evaluation with the JBI Critical Appraisal Tool [30].

According to the JBI Critical Appraisal Tool [30], twenty-five studies (published in 2002–2022), eleven studies (published in 2006–2023), and eighteen studies (published in 1998–2022) had a low, moderate (unclear), or high RoB, respectively (Table S7). Specific diagnostic histopathological criteria documenting “Patient selection” were provided in 31/54 (57.4%) studies. Detailed information regarding the demographic profile of patients and the site of lesions in all included groups of study, resulting in low RoB for the items of “Demographics” and “Clinical information”, were available in 35/54 (64,8%) and 38/54 (70.4%) studies, respectively. In those two items, the most common reason for unclear (moderate) RoB, which was observed in 14/54 (25.9%) studies for “Demographics” and in 9/54 (16.7%) for “Clinical information”, was the lack of relevant information for some of the study groups, usually that of the normal SG controls (Tables S3 and S7). The “Measure of the condition” item, corresponding to the adequate description of materials and methods applied for the immunohistochemical experiments, was classified as unclear (moderate) RoB in 40/54 (74.1%) studies, predominantly because of missing information regarding the negative and/or positive controls of immunohistochemistry (not available in 38 and 44 studies, respectively) or the antibodies’ dilution (not available in 19 studies, Tables S4 and S7). Similarly, the “Outcome” item was graded as unclear (moderate) RoB in 38/54 (70.4%) studies, mostly because of not specifying the cell types (e.g., ductal/myoepithelial or mucous/intermediate/epidermoid) that expressed the stem cell markers and/or the subcellular localization of staining (Tables S5 and S7). In contrast, the scoring system utilized for the evaluation of immunohistochemical findings and the statistical methods were mentioned in detail in 48/54 (88.9%) and 41/54 (75.9%) studies, resulting in a predominantly low RoB for the “Identification of the condition” and “Statistics” items (Tables S5 and S7). Figure 2 illustrates the RoB classification for each item in the JBI Critical Appraisal Tool [30].

### 3.7. Meta-Analysis

For each possible stem cell marker (i.e., c-KIT, CD44, CD133, and CD24) that was evaluated in multiple studies in the same disease pair (i.e., any pair of AdCC, MEC, PA, CXPA, P(LG)A, EMC, ACC, NOS, MYOC, LEP, SDC, and SCC), a meta-analysis was conducted on the studies that passed the selection criteria. Studies with no variation in test results across the two groups in each disease pair, i.e., studies with 100% positive cases for the marker of interest in both groups (specificity = zero) or studies with 100% negative cases for the marker of interest in both groups, were excluded from the meta-analysis. For AdCC, a meta-analysis was also performed for c-KIT on the solid AdCC versus cribriform/tubular AdCC histopathological variants. A pooled Diagnostic Odds Ratio (DOR) was calculated for all possible comparison pairs (Table S8), and all cases where the pooled DOR and both 95% confidence intervals were >1, or >0 and <1, were further examined. Results regarding pooled DORs for c-KIT, CD133, and CD44, and those concerning CD24 that did not produce any statistically significant results, are summarized in Table S8, while the studies included in the meta-analysis of each marker are presented in Tables S9–S12. Finally, meta-analyses were performed for the prognostic effect of the stem cell markers c-KIT, CD44, CD133, CD24, and NANOG in AdCC and MEC subgroups with advanced or small tumor sizes, AdCC subgroups with or without perineural invasion and metastasis; and in MEC subgroups with high/intermediate or low histopathological grade, without, however, concluding in any statistically significant results (Table S13).

#### 3.7.1. c-KIT

**Figure 3:**
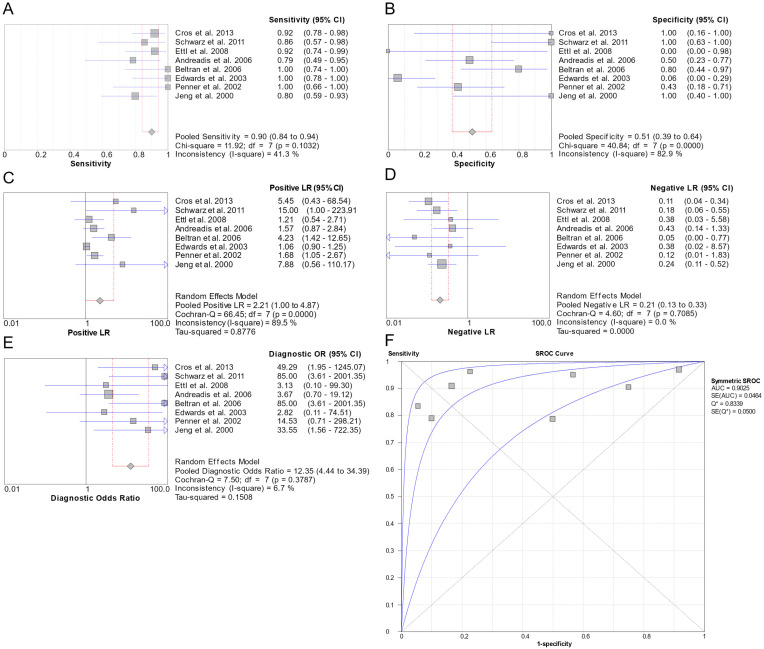
Forest plots of the pooled Sensitivity (**A**), Specificity (**B**), LR+ (**C**), LR- (**D**), DOR (**E**), and Summary Receiver Operating Characteristic (SROC) curve (**F**) of the eight studies involving the c-KIT for the AdCC and P(LG)A pair [60,65,72,75,76,78,79,80].

c-KIT was examined in multiple disease pairs (Tables S8 and S9). In the AdCC vs. P(LG)A comparison, a meta-analysis was conducted with eight eligible studies (Figure 3). Sensitivity was 0.90 (95% CI: 0.84–0.94), Specificity was 0.51 (95% CI: 0.39–0.64), Positive Likelihood Ratio (LR+) was 2.21 (95% CI: 1.00–4.87), Negative Likelihood Ratio (LR-) was 0.21 (95% CI: 0.13–0.33) and DOR was 12.35 (95% CI: 4.44–34.39). LR- had no heterogeneity between the studies (I^2^ = 0%), DOR had insignificant heterogeneity (I^2^ = 6.7%), and Sensitivity had low heterogeneity, with an I^2^ of 41.3%. Finally, Specificity and LR+ had high heterogeneity, with I^2^ of 82.9% and 89.5%, respectively. The diagnostic usefulness was excellent [Area Under the Curve (AUC) = 0.9025].

**Figure 4:**
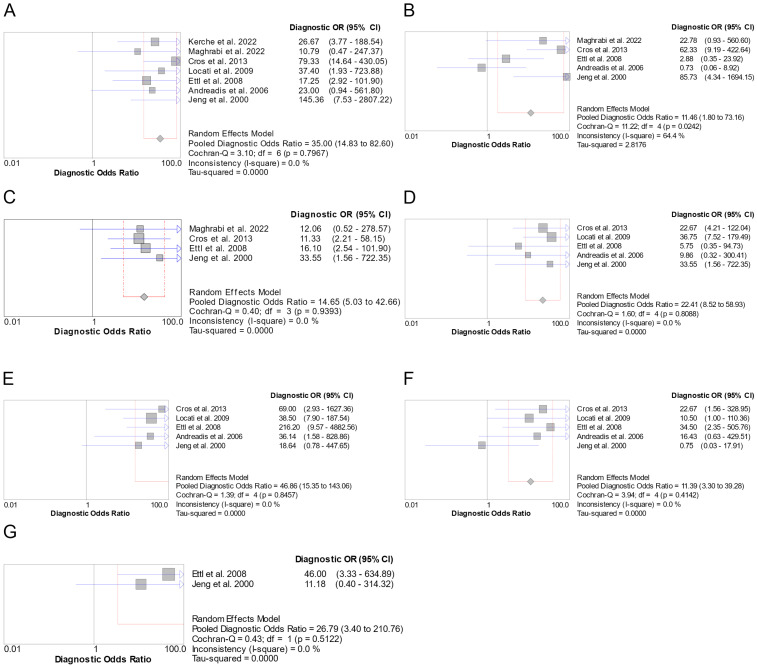
Forest plots of the pooled DOR involving c-KIT for the AdCC vs. MEC (**A**), ACC (**B**), CXPA (**C**), NOS (**D**), SDC (**E**), MYOC (**F**), and SCC (**G**) pairs [35,36,60,69,72,75,80].

There were seven studies included in the meta-analysis for AdCC vs. MEC. Sensitivity was 0.86 (95% CI: 0.80–0.91) (Figure S1A), Specificity was 0.77 (95% CI: 0.67–0.85) (Figure S1B), LR+ was 4.30 (95% CI: 1.39–13.30) (Figure S1C), LR- was 0.20 (95% CI: 0.14–0.29) (Figure S1D) and DOR was 35.00 (95% CI: 14.83–82.60) (Figure 4A). LR- and DOR showed no heterogeneity, Sensitivity had medium heterogeneity (I^2^ = 50.6%), while Specificity and LR+ presented high heterogeneity, with I^2^ of more than 80%. The discriminative ability was excellent (AUC = 0.9345) (Figure S1E).

There were five studies included in the meta-analysis for AdCC vs. ACC. Sensitivity was 0.89 (95% CI: 0.82–0.94) (Figure S2A), Specificity was 0.59 (95% CI: 0.43–0.73) (Figure S2B), LR+ was 1.86 (95% CI: 0.82–4.19) (Figure S2C), LR- was 0.22 (95% CI: 0.10–0.48) (Figure S2D) and DOR was 11.46 (95% CI: 1.80–73.16) (Figure 4B). LR- had low heterogeneity (I^2^ = 33.5%), while Sensitivity and DOR had medium heterogeneity, with I^2^ of 51.3% and 64.4%, respectively. Specificity and LR+ had high heterogeneity, with an I^2^ > 80%. The discriminative ability was excellent (AUC = 0.9247) (Figure S2E).

There were four studies included in the meta-analysis for AdCC vs. CXPA. Sensitivity was 0.91 (95% CI: 0.83–0.95) (Figure S3A), Specificity was 0.50 (95% CI: 0.33–0.67) (Figure S3B), LR+ was 1.74 (95% CI: 1.05–2.88) (Figure S3C), LR- was 0.19 (95% CI: 0.11–0.34) (Figure S3D) and DOR was 14.65 (95% CI: 5.03–42.66) (Figure 4C). LR- and DOR had no heterogeneity, while Sensitivity, LR+, and Specificity had medium heterogeneity, with I^2^ of 54.9%, 57.2%, and 69.2, respectively. The discriminative ability was good (AUC = 0.8951) (Figure S3E).

There were five studies included in the meta-analysis for AdCC vs. NOS. Sensitivity was 0.84 (95% CI: 0.77–0.89) (Figure S4A), Specificity was 0.81 (95% CI: 0.67–0.92) (Figure S4B), LR+ was 3.17 (95% CI: 1.32–7.62) (Figure S4C), LR- was 0.23 (95% CI: 0.16–0.33) (Figure S4D) and DOR was 22.41 (95% CI: 8.52–58.93) (Figure 4D). DOR and LR- showed no heterogeneity, Sensitivity had low heterogeneity (I^2^ = 26.2%), and Specificity and LR+ exhibited medium heterogeneity, with I^2^ of 53.7% and 55.8%, respectively. The discriminative ability was excellent (AUC = 0.9037) (Figure S4E).

There were five studies included in the meta-analysis for AdCC vs. SDC. Sensitivity was 0.84 (95% CI: 0.77–0.89) (Figure S5A), Specificity was 0.96 (95% CI: 0.85–0.99) (Figure S5B), LR+ was 9.09 (95% CI: 3.56–23.17) (Figure S5C), LR- was 0.21 (95% CI: 0.14–0.30) (Figure S5D) and DOR was 46.86 (95% CI: 15.35–143.06) (Figure 4E). Specificity, DOR, LR+, and LR- showed no heterogeneity, and Sensitivity had low heterogeneity, with an I^2^ of 26.2%. The discriminative ability was excellent (AUC = 0.9395) (Figure S5E).

There were five studies included in the meta-analysis for AdCC vs. MYOC. Sensitivity was 0.84 (95% CI: 0.77–0.89) (Figure S6A), Specificity was 0.67 (95% CI: 0.38–0.88) (Figure S6B), LR+ was 2.27 (95% CI: 0.75–6.86) (Figure S6C), LR- was 0.24 (95% CI: 0.14–0.40) (Figure S6D), and DOR was 11.39 (95% CI: 3.30–39.28) (Figure 4F). DOR showed no heterogeneity. Heterogeneity was insignificant for LR-, low for Sensitivity and Specificity (I^2^ of 26.2 and 36.3%, respectively), and medium for LR+ (I^2^ = 65.1). The discriminative ability was good (AUC = 0.8898) (Figure S6E).

There were two studies included in the meta-analysis for AdCC vs. SCC. Sensitivity was 0.86 (95% CI: 0.73–0.94) (Figure S7A), Specificity was 0.83 (95% CI: 0.36–1.00) (Figure S7B), LR+ was 4.03 (95% CI: 0.98–16.68) (Figure S7C), LR- was 0.18 (95% CI: 0.06–0.54) (Figure S7D), and DOR was 26.79 (95% CI: 3.40–210.76) (Figure 4G). Specificity, DOR, and LR+ showed no heterogeneity, while Sensitivity and LR- had low heterogeneity, with I^2^ of 35% and 35.1%, respectively. As there were only two studies included, an SROC curve was not produced, and thus, AUC was not calculated.

The discriminative ability of c-KIT was also examined between the solid and cribriform/tubular variants of AdCC in a meta-analysis that included six studies (Figure S8). However, as the pooled DOR was 2.10 (95% CI: 0.55–8.01), it was not considered statistically significant due to the confidence interval including 1.

Furthermore, meta-analyses were conducted for c-KIT in the comparison of EMC with MEC, P(LG)A, NOS, MYOC, and SDC. As there were only two studies involved in each comparison, SROC curves could not be produced, and thus, no AUC was calculated.

In the meta-analysis for EMC vs. MEC, Sensitivity was 0.89 (95% CI: 0.52–1.00) (Figure S9A), Specificity was 0.89 (95% CI: 0.71–0.98) (Figure S9B), LR+ was 6.72 (95% CI: 2.40–18.77) (Figure S9C), LR- was 0.17 (95% CI: 0.04–0.76) (Figure S9D), and DOR was 38.16 (95% CI: 4.56–319.29) (Figure S9E). All pooled metrics showed no heterogeneity.

In the meta-analysis for EMC vs. P(LG)A, Sensitivity was 0.89 (95% CI: 0.52–1.00) (Figure S10A), Specificity was 0.56 (95% CI: 0.30–0.80) (Figure S10B), LR+ was 1.86 (95% CI: 0.99–3.48) (Figure S10C), LR- was 0.26 (95% CI: 0.07–0.94) (Figure S10D), and DOR was 10.69 (95% CI: 1.03–111.36) (Figure S10E). Sensitivity, LR-, and DOR showed no heterogeneity, LR+ had insignificant heterogeneity (I^2^ = 0.8%), and Specificity had medium heterogeneity (I^2^ = 60.3%).

In the meta-analysis for EMC vs. NOS, Sensitivity was 0.89 (95% CI: 0.52–1.00) (Figure S11A), Specificity was 0.69 (95% CI: 0.39–0.91) (Figure S11B), LR+ was 2.60 (95% CI: 1.14–5.93) (Figure S11C), LR- was 0.22 (95% CI: 0.05–1.01) (Figure S11D), and DOR was 11.96 (95% CI: 1.40–101.92) (Figure S11E). All pooled metrics showed no heterogeneity.

In the meta-analysis for EMC vs. MYOC, Sensitivity was 0.89 (95% CI: 0.52–1.00) (Figure S12A), Specificity was 0.80 (95% CI: 0.28–0.99) (Figure S12B), LR+ was 3.10 (95% CI: 0.078–12.34) (Figure S12C), LR- was 0.21 (95% CI: 0.04–1.01) (Figure S12D) and DOR was 15.81 (95% CI: 1.22–205.57) (Figure S12E). Sensitivity, LR+, LR-, and DOR showed no heterogeneity, and Specificity had insignificant heterogeneity (I^2^ = 15.6%)

In the meta-analysis for EMC vs. SDC, Sensitivity was 0.89 (95% CI: 0.52–1.00) (Figure S13A), Specificity was 1.00 (95% CI: 0.63–1.00) (Figure S13B), LR+ was 8.08 (95% CI: 1.14–52.62) (Figure S13C), LR- was 0.21 (95% CI: 0.06–0.76) (Figure S13D), and DOR was 40.37 (95% CI: 2.82–577.21) (Figure S13E). All pooled metrics showed no heterogeneity.

#### 3.7.2. CD133

**Figure 5:**
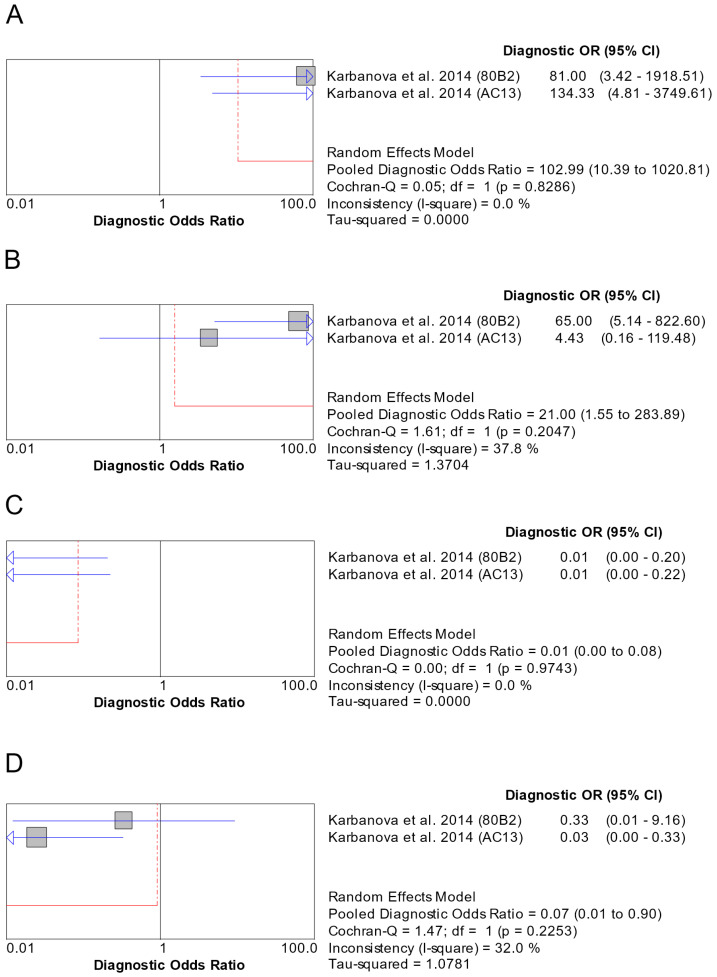
Forest plots of the pooled DOR involving CD133 for the AdCC vs. MEC (**A**), ACC vs. MEC (**B**), MEC vs. PA (**C**), and ACC vs. PA (**D**) pairs [58].

CD133 was examined in multiple disease pairs (Tables S8 and S10). Statistically significant results are marked in bold in Table S8 and depicted in Figure 5. As there were only two studies [52,58] (or two experiments included in the same study [58]) involved in each comparison that met the eligibility criteria for meta-analysis, SROC curves could not be produced, and thus, no AUC was calculated.

In the meta-analysis for AdCC vs. MEC, Sensitivity was 0.93 (95% CI: 0.66–1.00) (Figure S14A), Specificity was 0.93 (95% CI: 0.78–0.99) (Figure S14B), LR+ was 7.82 (95% CI: 2.35–26.03) (Figure S14C), LR- was 0.16 (95% CI: 0.04–0.56) (Figure S14D), and DOR was 102.99 (95% CI: 10.39–1020.81) (Figure 5A). LR- and DOR presented no heterogeneity, LR+ had insignificant heterogeneity (I^2^ = 8.9%), Sensitivity had low heterogeneity (I^2^ = 31.7%), and Specificity had medium heterogeneity (I^2^ = 65.7%).

In the meta-analysis for ACC vs. MEC, Sensitivity was 0.50 (95% CI: 0.28–0.72) (Figure S15A), Specificity was 0.93 (95% CI: 0.78–0.99) (Figure S15B), LR+ was 6.30 (95% CI: 1.89–20.96) (Figure S15C), LR- was 0.32 (95% CI: 0.00–25.32) (Figure S14D), and DOR was 21.00 (95% CI: 1.55–283.89) Figure 5B). LR+ presented no heterogeneity, DOR had low heterogeneity (I^2^ = 37.8%), Specificity had medium heterogeneity (I^2^ = 65.7%), and Sensitivity and LR- had high heterogeneity (I^2^ of 94.2% and 95.3%, respectively).

In the meta-analysis for MEC vs. PA, Sensitivity was 0.07 (95% CI: 0.01–0.22) (Figure S16A), Specificity was 0.10 (95% CI: 0.01–0.32) (Figure S16B), LR+ was 0.13 (95% CI: 0.05–0.38) (Figure S16C), LR- was 5.68 (95% CI: 1.61–20.08) (Figure S16D) and DOR was 0.01 (95% CI: 0.00–0.08) Figure 5C). LR+ and DOR presented no heterogeneity, LR- had insignificant heterogeneity (I^2^ = 15.5%), and Sensitivity and Specificity had medium heterogeneity (I^2^ of 65.7% and 66.6%, respectively).

In the meta-analysis for ACC vs. PA, Sensitivity was 0.50 (95% CI: 0.28–0.72) (Figure S17A), Specificity was 0.10 (95% CI: 0.01–0.32) (Figure S17B), LR+ was 0.34 (95% CI: 0.01-17.13) (Figure S17C), LR- was 4.23 (95% CI: 1.32–13.53) (Figure S17D), and DOR was 0.07 (95% CI: 0.01–0.90) (Figure 5D). LR- presented no heterogeneity, DOR had low heterogeneity (I^2^ = 32.0%), Specificity had medium heterogeneity (I^2^ = 66.6%), and Sensitivity and LR+ had high heterogeneity (I^2^ of 94.2% and 94.1%, respectively).

#### 3.7.3. CD44

CD44 was examined in multiple disease pairs (Tables S8 and S11). In the meta-analysis of AdCC and PA comparison that comprised three studies, Sensitivity was 0.67 (95% CI: 0.43–0.85) (Figure S18A), Specificity was 0.09 (95% CI: 0.02-0.20) (Figure S18B), LR+ was 0.77 (95% CI: 0.56–1.06) (Figure S18C), LR- was 4.21 (95% CI: 1.47–12.02) (Figure S18D), and DOR was 0.15 (95% CI: 0.04–0.62) (Figure S18E). Specificity, LR-, and DOR showed no heterogeneity, LR+ had insignificant heterogeneity (I^2^ = 14.8%), and Sensitivity had low heterogeneity (I^2^ = 30.7%). The AUC was 0.1347.

## 4. Discussion

Herein, a thorough systematic review of the stem cell immunohistochemical signature in MSGTs is provided for the first time, accompanied by meta-analyses that highlighted c-KIT as a potential discriminative marker among MSGTs.

KIT Proto-Oncogene, Receptor Tyrosine Kinase (KIT, also known as C-KIT or CD177) is involved in crucial biological processes, such as cell proliferation, differentiation, and survival, and has been implicated in various malignancies, e.g., gastrointestinal stromal tumors, breast cancer, thyroid cancer, and colorectal cancer; thus, its potential targeting for therapeutic reasons is attracting clinical and research interest [82,83]. The present study summarized the scientific evidence supporting c-KIT overexpression in AdCC. The most common genetic alteration in AdCC is the fusion of the *MYB* gene to the transcription factor *NFIB* by a translocation t(6; 9) (q22–23; p23–24) that leads to sustained Myb protein stability [84], which regulates c-KIT on a transcriptional level [85]. Therefore, c-KIT expression in AdCC may result from *MYB* fusion, as the presence of activating mutations of c-KIT in AdCC is controversial [86]. Although the successful management of AdCC cases with a selective tyrosine kinase inhibitor of c-KIT imatinib has been reported [87], as imatinib mesylate is efficacious in tumors with activating *KIT* mutations [86]. Further research is warranted to elucidate the therapeutic effect of this molecule on AdCC.

A remarkable finding of the meta-analysis was the outstanding discriminatory power of c-KIT in AdCC versus P(LG)A. Given the considerable clinicopathological overlap between these two MSGTs [88], c-KIT could facilitate their differential diagnosis. The meta-analysis also showed statistically significant differences in the expression of c-KIT in EMC, in which it was overexpressed, compared to other tumors, but the small number of available studies limited the possibility of drawing a conclusion about the discriminatory power of the marker. It should be noted that the more common molecular aberrations in EMCs are mutations in *HRAS* [84], a molecule that functions as a switch in the RAS/MAPK signaling pathway activated by c-KIT. Therefore, the mechanism underlying c-KIT overexpression in EMC needs to be clarified. The common finding of c-KIT expression in most cases of AdCC and EMC might be associated with the shared biphasic histopathological pattern (i.e., including ductal and myoepithelial cells) of those tumors that may occasionally hinder their distinction. Further studies based on advanced molecular techniques, e.g., next-generation sequencing, are required to explore if additional molecular similarities exist between AdCC and EMC.

Meta-analysis also revealed significantly reduced expression of CD133 in MEC compared to AdCC, ACC, or PA. CD133, also known as prominin-1, is a transmembrane glycoprotein that exerts a significant role in maintaining cellular organization, promoting asymmetric cell division, and supporting self-renewal and differentiation potential [89]. CD133 is expressed in normal salivary glands and neoplasms of the salivary gland [58]. As meta-analysis results for CD133 were based on the immunohistochemical experiments conducted with two different anti-CD133 antibodies by one research group [58], they should be considered with caution.

Various studies included in the qualitative analysis pointed out the high expression of CD44 in different MSGTs, as well as in BSGTs and normal salivary glands (Table S5), while meta-analysis revealed significantly decreased CD44 expression in AdCC than in PA (Figure S18). CD44 (Cluster of differentiation 44) is a transmembrane glycoprotein encoded by the CD44 gene that contributes to the maintenance of cell proliferation, differentiation, and survival and has been associated with the aggressiveness of several human cancers [90,91]. Moreover, CD44 acts as a receptor for hyaluronan and other components of the extracellular matrix, thus promoting cell adhesion and migration, which are vital processes in tissue regeneration [92]. Further studies are needed to investigate the possible involvement of CD44 in the regeneration of salivary glands, for example, in cases of radiation injury.

Another marker included in the quantitative analysis was CD24. CD24 (Cluster of differentiation 24) is a highly glycosylated, small-cell adhesion protein with a principal role in cell–cell communication, cell–matrix interaction, and immune system regulation [93]. As a marker of stemness, CD24 promotes proliferation, migration, and invasion and has been implicated in tumorigenesis by representing a so-called “don’t eat me” signal on the tumor cell surface, thus inhibiting their phagocytosis by macrophages and evading the cytotoxic action of Natural Killer cells [94,95]. Three studies reported CD24 expression in various MSGTs, BSGTs, as well as in normal salivary glands [22,39,54], although meta-analysis did not reveal any statistically significant results. As recent studies suggest that CD24 may be a potential therapeutic target in cancer [95,96], the role of CD24 in SGTs warrants further investigation.

As shown in Table S5, a lot of immunohistochemical studies suggested the prognostic significance of stem cell marker expression in MSGTs. However, no significant associations were revealed in the meta-analysis, probably because of the small number of eligible studies. Additional studies reporting the correlation of stem cell markers’ expression with prognostic variables of different types of MSGTs should further evaluate the usefulness of stem cell markers in guiding the prognosis of MSGTs.

A point of strength of the current study is that for the first time, a comprehensive literature search regarding stem cell markers’ immunohistochemical profile in MSGTs was performed, and all available evidence was summarized and critically appraised. Moreover, no exclusion criterion regarding the anatomic location of salivary gland tumors was applied, in contrast to previous studies focusing on the parotid gland that represents the primary site of salivary gland malignancies [97], in order to maximize the available number of eligible studies. In addition, most of the included studies presented a low risk of bias, a finding that strengthens the reliability of the evidence provided in this systematic review. However, missing information regarding the immunohistochemical method, i.e., positive/negative controls and antibody dilutions, in a considerable number of studies, as well as significant variability in the antibodies used against the same marker or the dilutions of the same antibody was observed, which hinders the direct comparison of results and might account for significant differences between studies. Another drawback of the present study is the small number of rare MSGTs included, which limits the ability to draw reliable conclusions regarding their stem cell immunohistochemical phenotypes.

## 5. Conclusions

This systematic review summarized, for the first time, the pertinent literature on stem cell immunoexpression on MSGTs, providing insights into the possible link between stem cells and the histopathological heterogeneity and diverse biological behaviors that characterize salivary gland malignancies. The qualitative synthesis outlined the immunoexpression pattern of the stem cell markers ABCG2, ALDH1, BMI1, CD10, CD24, CD44, CD133/PROM1, CD166, c-KIT/CD117, DCLK1, EZH2, NANOG, OCT4/POU5F1, and SOX2 in the parenchyma and/or stroma of various MSGTs. The meta-analysis highlighted the excellent discriminative ability of c-KIT between AdCC and P(LG)A, which may facilitate their discrimination in diagnostically challenging cases, and revealed statistically significant c-KIT overexpression in EMC compared with other MSGTs. Finally, the quantitative analysis of a few eligible studies revealed underexpression of CD133 and CD44 in MEC and AdCC, respectively, findings that require further investigation and validation through additional studies.

## Figures and Tables

**Table 1:** Number (N) of cases in each MSGT, BSGT, and control group (Population), stem cell markers (Comparators), and expression results (Outcome-1) in the 54 studies included in the qualitative analysis.

Study No.	Reference	MSGTs; BSGTs; Controls (N)	Marker	Outcome of Expression
1	Santos et al., 2023 [21]	MEC (20), AdCC (20); PA (20); normal SGs (4)	CD44, ALDH1, OCT4, SOX2	A significantly higher ALDH1 parenchymal expression was observed in PA than AdCC (*p* < 0.001) or MEC (*p* = 0.006) and in MEC than AdCC (*p* < 0.001). In contrast, a significantly higher number of positive stromal cases was observed among MEC and AdCC groups than among PA (*p* = 0.004). Stromal expression of CD44 was significantly higher in MEC and AdCC compared with PA (*p* = 0.003). A significantly higher OCT4 parenchymal expression was observed in AdCC than in MEC (*p* = 0.001) or PA (*p* = 0.004). A significantly higher OCT4 stromal expression was observed in AdCC and MEC compared with PA (*p* < 0.001). A significantly higher SOX2 parenchymal expression was observed in MEC than in AdCC (*p* < 0.001) or PA (*p* < 0.001).
2	Kerche et al., 2022 [35]	MEC (14), AdCC (18); PA (23); non-neoplastic SG lesions (10)	c-KIT	A significantly lower expression of c-KIT was observed in MEC compared with AdCC and PA (*p* < 0.001).
3	Maghrabi et al., 2022 [36]	MEC (11), AdCC (20), CXPA (10), ACC (6), P(LG)A (3)	c-KIT	A significant difference in c-KIT expression was observed between the studied MSGTs, with AdCC and P(LG)A being the most common strong positive cases (*p* < 0.05). No comparison between ACC or MEC subgroups.
4	Marleen et al., 2022 [37]	MEC [34; low grade (13), moderate grade (3), high grade (18)]	CD44	An increased CD44 expression H-score was found in low-grade MEC compared to high/intermediate-grade MEC (*p* = 0.05).
5	Sadeghi et al., 2022 [38]	MEC (48), AdCC (47)	CD44, CD133, SOX2	A significantly higher number of cases with high CD133 expression were observed in the AdCC group compared with the MEC group (*p* = 0.001). No difference in CD44 and SOX2 expression.
6	Yildirim & Shuibat, 2022 [39]	MEC (6), AdCC (8), ACC (2), CXPA (2), P(LG)A (6); PA (21), BCA (3); normal SGs (7)	ALDH1, CD24, CD44, CD166	A significantly decreased number of ALDH1 positive cases (*p* = 0.034) and a significantly increased number of CD166 positive cases (*p* = 0.002) were found in the malignant SGT group compared to benign SGTs and normal SGs.
7	Bertlich et al., 2021 [40]	SCC [31; primary (11), metastatic (13), NA (7)]	ALDH1, BMI1, CD44, SOX2	CD44 and ALDH1 1 may be useful in differentiating between primary and metastatic SCC.
8	Moura et al., 2021 [41]	MEC (20), AdCC (20); PA (20); normal SGs (10)	OCT4, CD44	A significantly higher OCT4 staining score was observed in MEC and AdCC compared with PA (*p* = 0.001). No significant difference in CD44 staining score.
9	da Silva et al., 2020 [42]	103 MSGTs[MEC (25), ACC (15), AdCC (13), P(LG)A (10), NOS (13), EMC (8), CXPA (7), SDC (5), BCAC(4), ClCC (3)]; 51 BSGTs [PA (25), MYO (9), WT (7), CAD (5), BCA (5)]	ALDH1	A significantly increased ALDH1 stromal high expression was found in MSGTs (57/103, 55.3%) than in BSGTs (11/51, 21.6%) (*p* < 0.0001). No statistically significant difference among the various MSGTs was mentioned.
10	Spiegel et al., 2020 [20]	MEC (5), AdCC (6), ACC (1), NOS (11), P(LG)A (3), CXPA (5), ΕΜC (2), MYOC (1), SDC (6)	ALDH1, BMI1, CD44, NANOG, SOX2	No comparison of ALDH1, BMI1, CD44, NANOG, and SOX2 expression between various malignant SGTs was provided.
11	Esmaeil et al., 2019 [43]	MEC [80; low grade (28), intermediate grade (16), high grade (36)]; normal SGs (10)	CD44	CD44 expression, in terms of the percentage of positive cells as well as staining intensity, was significantly correlated with histopathological grade (*p* < 0.001), with high-grade MEC showing the most increased CD44 expression.
12	Luo et al., 2019 [44]	AdCC [34; cribriform/tubular (26), solid (8)]; normal SGs (10)	SOX2	SOX2 expression was detected in a significantly higher number of AdCC cases than normal SGs (*p* < 0.05). SOX2 expression was detected in a significantly higher number of AdCC cases with a solid pattern (7/8) than a tubular/cribriform pattern (9/26, *p* = 0.014).
13	Tsai et al., 2019 [45]	AdCC [38; cribriform/tubular (15), solid (6)]	c-KIT	c-KIT expression was observed in 40.9% of cribriform cases and 100% of tubular and solid cases. No statistical comparison of the expression results was provided.
14	Destro Rodrigues et al., 2017 [23]	MEC [28; low grade (21), intermediate grade (4), high grade (3)]; normal SGs from same patients (20)	CD44, OCT4, BMI1, NANOG	No significant difference in all markers’ expression between MEC and normal SGs.
15	Kadletz et al., 2017 [46]	80 MSGTs [MEC (10), AdCC (26), ACC (8), NOS (17), CXPA (5), BCAC (3), ClCC (2), NEC (4), SCC (5)]	DCLK1	No significant difference.
16	Sedassari et al., 2017 [47]	CXPA (25); PA (30)	SOX2	CXPA were 96% positive, while PA were 100% negative (*p* < 0.00001).
17	Xu et al., 2017 [48]	MEC [75; low grade (38), intermediate grade (31), high grade (6)]	CD44, CD133, SOX2, NANOG	No significant difference between the different histopathological grades was observed in CD44 (*p* = 0.829), CD133 (*p* = 0.556), SOX2 (*p* = 0.592), or NANOG (*p* = 0.803) expression.
18	Binmadi et al., 2016 [49]	MEC [15; low grade (5), intermediate grade (2), high grade (8)]	CD44	No significant difference between the different histopathological grades of MEC.
19	Jain et al., 2016 [50]	AdCC [30; cribriform (10), tubular (10), solid (10)]	c-KIT	The mean number of c-KIT-positive cells was significantly higher in the solid pattern of AdCC (83.50 ± 7.53) compared with the tubular pattern (73.90 ± 8.93) and the cribriform pattern (73.20 ± 6.61, *p* < 0.05).
20	Seifi et al., 2016 [51]	MEC (15), AdCC (15); PA (15)	ALDH1	No difference in the number of positive cases between MEC, AdCC, PA (p>0.05), or between low- and high-grade MEC (*p* > 0.05), or between the different histopathologic subtypes of AdCC (*p* > 0.05).
21	Wang et al., 2016 [52]	AdCC (45); PA (20); normal SGs (10)	CD133	CD133 expression was significantly associated with the AdCC group (46.67% positive) compared with PA or normal SG groups that were both 100% negative (*p* < 0.05). Among AdCC cases, a significantly higher number of positive cases was observed in cases with a solid pattern (12/13) than with a tubular or cribriform pattern (9/32, *p* < 0.0001).
22	Yi et al., 2016 [53]	AdCC [102; cribriform/tubular (86), solid (16)]; normal SGs (10)	BMI1	No significant difference between the different histopathological subtypes was observed in BMI1 expression (*p* = 0.621). No statistical comparison of the expression results of AdCC and normal SGs was provided.
23	Adams et al., 2015 [54]	MEC [12; cystic (3), mixed (3), solid (6)]; normal SGs (NA)	ALDH1, CD10, CD24, CD44	More intense expression of ALDH1, CD10, CD24, and CD44 in MEC than normal SGs. ALDH1, CD10, and CD24 were expressed more in solid than cystic MECs.
24	Azúa-Romeo et al., 2014 [55]	MEC (5), AdCC (5), P(LG)A (3), ACC (4), NOS (3), SCC (9), ClCC (2), sarcoma (1); PA (17), WT (6), Oncocytoma (2); non-neoplastic SG lesions (9)	CD44	A significantly higher CD44 score was observed in MSGTs compared with BSGTs or normal SGs (*p* < 0.01).
25	Chang et al., 2014 [24]	AdCC (50), normal SGs (20)	BMI1	A significantly increased BMI1 expression was found in AdCC than in normal SGs (*p* < 0.01).
26	Dai et al., 2014 [56]	AdCC [131; cribriform/tubular (102), solid (29)]; normal SGs from the same patients (NA)	SOX2	Increased SOX2 expression in AdCC tissue compared to normal SGs (finding not supported by statistical analysis result). No difference between AdCC histopathological subtypes.
27	Fok et al., 2014 [57]	MEC (29), ACC (11); PA (20); normal SGs (20)	CD44	A significantly higher CD44 score was observed in ACC compared with MEC (*p* < 0.0001), PA (*p* < 0.0001), and normal SGs (*p* < 0.0001).
28	Karbanova et al., 2014 [58]	MEC (15), AdCC (7), ACC (11); PA (10); non-neoplastic SGs (3)	CD133	No statistical comparison of the expression results was provided.
29	Li et al., 2014 [12]	AdCC [25; cribriform (16), cribriform+ solid (4), cribriform with tubular (5)]; normal SGs (10)	CD133, ABCG2	Significantly stronger ABCG2 expression was observed in AdCC than in normal SGs (*p* = 0.000). No significant difference in CD133 expression was noted between AdCC and normal SGs.
30	Phuchareon et al., 2014 [59]	AdCC [27; cribriform (3), tubular (4), solid (1), cribriform+tubular (10), solid+tubular (8), cribriform+solid (1)]; normal SGs from the same patients (5)	c-KIT	No statistical comparison of the immunoexpression results was provided (AdCC vs. normal SGs). All AdCC histopathological subtypes were 100% positive, but differences in intensity were not statistically compared.
31	Cros et al., 2013 [60]	MEC (30), AdCC (40), ACC (14), P(LG)A (2), CXPA (13), NOS (12), EMC (6), MYOC (3), SDC (3)	c-KIT	No statistical comparison of c-KIT expression between various malignant SGTs.
32	Fok et al., 2013 [61]	AdCC (22), P(LG)A (16); PA (20); normal SGs (20)	CD44	A significantly higher CD44 score was observed in normal SGs than in AdCC (*p* < 0.0002), P(LG)A (*p* = 0.04), and PA (*p* < 0.001).
33	Soave et al., 2013 [22]	MEC (11), AdCC (34), ACC (4), NOS (5), P(LG)A (3), CXPA (4), BCAC (5), SDC (3)	CD24, CD44	No statistical comparison of the expression results was provided.
34	Zhou et al., 2013 [62]	AdCC [184; cribriform (103), tubular (52), solid (29)]	ALDH1	No significant difference in ALDH1 expression between AdCC histopathological patterns.
35	Fujita & Ikeda, 2012 [14]	AdCC [26; cribriform (26/26), tubular (22/26), solid (6/26)]	CD44, CD133	No significant difference in CD133 or CD44 expression between AdCC histopathological patterns.
36	Lee et al., 2012 [63]	AdCC [48; cribriform (18), tubular (2), solid (7), indeterminate (21)]	c-KIT	No significant difference in c-KIT expression between AdCC histopathological patterns.
37	Kim et al., 2011 [64]	CXPA [17; luminal (11), non-luminal (6)]	c-KIT	No significant difference in c-KIT expression between luminal and non-luminal CXPA.
38	Schwarz et al., 2011 [65]	AdCC (14), P(LG)A (8)	c-KIT	The mean percentage of c-KIT-positive cells was significantly higher in ACC (37.9%) than P(LG)A (3.1%, *p* < 0.001).
39	Bell et al., 2010 [66]	AdCC [199; cribriform (109), tubular (57), solid (24), ΝA (9)]	c-KIT	A significantly higher number of ductal c-KIT expression was observed in solid than cribriform/tubular pattern of AdCC (*p* = 0.00003).
40	Tang et al., 2010 [67]	AdCC [121; cribriform/tubular (85/121), solid (36/121)]	c-KIT	A significantly higher number of positive cases was observed in cases with a solid pattern (36/36) than with a tubular or cribriform pattern (72/85, *p* = 0.02).
41	Tetsu et al., 2010 [68]	AdCC [17; cribriform (7), tubular (4), solid (1), tubular+cribriform (4), solid+ cribriform (1)]; normal SGs from the same patients (NA)	c-KIT	No statistical comparison of the expression results was provided. A total of 15 out of 17 cases were positive. Two cases (1 cribriform, 1 cribriform + tubular) were negative.
42	Locati et al., 2009 [69]	MEC (5), AdCC (63), NOS (26), SDC (26), MYOC (5), Poorly differentiated Ca (4), other histopathological type adenocarcinomas (10)	c-KIT	78% AdCC, 25% MYOC, and 8–10% of NOS, SDC, and other histopathological type adenocarcinomas were c-KIT positive, whereas MEC and poorly differentiated Ca were negative. No statistical comparison of the expression results was provided.
43	Sequeiros-Santiago et al., 2009 [70]	AdCC [24; cribriform (18), tubular (3), solid (3)]	c-KIT	No significant difference in c-KIT expression was observed between the AdCC histopathological subtypes (*p* = 0.367).
44	Vila et al., 2009 [71]	AdCC [14; cribriform (5), cribriform/tubular (3), cribriform/tubular+solid (4), solid (2)]	c-KIT	All AdCC histopathological subtypes were 100% positive.
45	Ettl et al., 2008 [72]	MEC (15), AdCC (25), ACC (11), P(LG)A (1), CXPA (13), NOS (3), MYOC (4), SDC (12), BCAC (3), SCC (5), LEP (3), OncoCa (3), CystadenoCa (1), Undifferentiated Ca (1), Adenosquamous Ca (1)	c-KIT	Significantly high numbers of positive and negative cases of c-KIT were observed in AdCC (*p* = 0.001) and SDC (*p* = 0.001) groups, respectively, compared with other malignant SGTs.
46	Vekony et al., 2008a [73]	MYOC (30); MYO (54); normal SGs (17)	BMI1, EZH2	A significantly higher percentage of EZH2-immunopositive cells were observed in MYOC than in MYO (*p* = 0.005). No significant difference in the percentage of BMI1-immunopositive cells was observed in MYOC than in MYO.
47	Vekony et al., 2008b [74]	AdCC [21; cribriform/tubular (15), solid (6)]; normal SGs from the same patients (17)	BMI1, EZH2	No significant difference in BMI1 and EZH2 expression between AdCC and normal SGs. No available comparison of AdCC subtypes.
48	Andreadis et al., 2006 [75]	MEC (3), AdCC (14), P(LG)A (14), ACC (6), SDC (3), EMC (3), Carcinosarcoma (1), NOS (1), LEP (3), SCAC (5), MYOC (2), Oncocytic Ca (1), BCAC (1); PA (20), WT (15), MYO (2), ONC (2), BCA (1); non-neoplastic SG lesions(5)	c-KIT	c-KIT is expressed in both benign and malignant SGTs, as well as in non-neoplastic SG disease.
49	Beltran et al., 2006 [76]	AdCC (12), P(LG)A (20)	c-KIT	A significantly higher expression score was observed in AdCC compared with P(LG)A (*p* < 0.001).
50	Perschbacher et al., 2004 [77]	MEC (4), AdCC (22), P(LG)A (13), NOS (8), ACC (6); PA (5); normal SGs (30)	c-KIT	No significant differences.
51	Edwards et al., 2003 [78]	AdCC (15), P(LG)A (17); MAD (17)	c-KIT	No difference in the number of positive cases. Staining intensity was stronger in ACC than P(LG)A (statistical analysis not performed).
52	Penner et al., 2002 [79]	AdCC (9), P(LG)A (14)	c-KIT	No statistical comparison of the expression results was provided.
53	Jeng et al., 2000 [80]	MEC (19), AdCC (25), ACC (11), P(LG)A (4), CXPA (4), NOS (4), MYOC (2), SDC (2), SCC (1), LEP (6), Undif.Ca (1)	c-KIT	A statistically significant number of positive cases for c-KIT was observed in AdCC, LEC, and MYOC groups compared with other MSGTs that are all negative (*p* < 0.00001).
54	Xing et al., 1998 [81]	MEC (10), AdCC (8), P(LG)A (10), PA (9), MAD (9)	CD44	CD44 was expressed in 100% of MEC, 87.5% of AdCC, 80% of P(LG)A, 100% of PA, and 11.1% of MAD. No statistical comparison of the expression results was provided.

Abbreviations: ACC, acinic cell carcinoma; AdCC: adenoid cystic carcinoma; Adenosquamous Ca, adenosquamous carcinoma; BCA, basal cell adenoma; BCAC, basal cell adenocarcinoma; BSGTs, benign salivary gland tumors; CAD, canalicular adenoma; ClCC, clear cell carcinoma, CXPA, carcinoma ex pleomorphic adenoma; CystadenoCa, cystadenocarcinoma; EMC, epithelial–myoepithelial carcinoma; LEP; lymphoepithelial carcinoma; MAD, monomorphic adenoma; MEC, mucoepidermoid carcinoma; MSGTs, malignant salivary gland tumors, MYOC, myoepithelial carcinoma; MYO, myoepithelioma; NA, not available; NEC, neuroendocrine carcinoma; NOS, not otherwise specified adenocarcinoma; OncoCa, oncocytic carcinoma; PA, pleomorphic adenoma; P(LG)A, polymorphous low-grade adenocarcinoma; poorly differentiated Ca, poorly differentiated carcinoma; SCC, squamous cell carcinoma; SDC, salivary duct carcinoma; SGs, salivary glands; SGTs, salivary gland tumors; Undif.Ca, undifferentiated carcinoma; WT, Warthin’s tumor.

## Data Availability

The original contributions of the study are included in the article and Supplementary Materials. Further inquiries can be directed to the corresponding author.

## Supplementary Materials

The following supporting information can be downloaded at: https://www.mdpi.com/article/10.3390/genes16010037/s1, Table S1. Keywords applied for the literature search in each scientific database; Table S2. Records excluded from qualitative analysis and reasons for exclusion at full-text evaluation phase; Table S3. Journal of publication, disease and control groups, sample type, number of patients, demographic characteristics, site of samples, documentation of disease/control entities’ diagnosis, and histopathologic variants in case of solid ameloblastomas; Table S4. Stem cell markers used, details of the immunohistochemical experiments, and the method for staining evaluation; Table S5. Outcome of comparison of the expression and prognostic significance of stem cell markers between MSGTs, BSGTs, and normal SGs or non-neoplastic SG lesions; Table S6. Interpretation of the Critical Appraisal Tool proposed by the JBI [30] for RoB assessment in the present study; Table S7. RoB evaluation results per study using the JBI [30] Critical Appraisal Tool; Table S8. Pooled DOR for all possible comparison pairs; Table S9. All possible comparison pairs for DOR of c-KIT; Table S10. All possible comparison pairs for DOR of CD133; Table S11. All possible comparison pairs for DOR of CD44; Table S12. All possible comparison pairs for DOR of CD24; Table S13. Pooled Prognostic OR for all possible comparison pairs; Figure S1: Meta-analysis for c-KIT in AdCC vs. MEC; Figure S2: Meta-analysis for c-KIT in AdCC vs. ACC; Figure S3: Meta-analysis for c-KIT in AdCC vs. CXPA; Figure S4: Meta-analysis for c-KIT in AdCC vs. NOS; Figure S5: Meta-analysis for c-KIT in AdCC vs. SDC; Figure S6: Meta-analysis for c-KIT in AdCC vs. MYOC; Figure S7: Meta-analysis for c-KIT in AdCC vs. SCC; Figure S8: Meta-analysis for c-KIT in solid AdCC vs. cribriform/tubular AdCC; Figure S9: Meta-analysis for c-KIT in EMC vs. MEC; Figure S10: Meta-analysis for c-KIT in EMC vs. P(LG)A; Figure S11: Meta-analysis for c-KIT in EMC vs. NOS; Figure S12: Meta-analysis for c-KIT in EMC vs. MYOC; Figure S13: Meta-analysis for c-KIT in EMC vs. SDC; Figure S14: Meta-analysis for CD133 in AdCC vs. MEC; Figure S15: Meta-analysis for CD133 in ACC vs. MEC; Figure S16: Meta-analysis for CD133 in MEC vs. PA; Figure S17: Meta-analysis for CD133 in ACC vs. PA; Figure S18: Meta-analysis for CD44 in AdCC vs. PA.

## Abbreviations

ACC, acinic cell carcinoma; AdCC, adenoid cystic carcinoma; Adenosquamous Ca, adenosquamous carcinoma; AUC, Area Under the Curve; BCA, basal cell adenoma; BCAC, basal cell adenocarcinoma; BSGTs, benign salivary gland tumors; CAD, canalicular adenoma; ClCC, clear cell carcinoma, CSCs, cancer stem cells; CXPA, carcinoma ex pleomorphic adenoma; CystadenoCa, cystadenocarcinoma; DOR, Diagnostic Odds Ratio; EMC, epithelial–myoepithelial carcinoma; FFPE, formalin-fixed, paraffin-embedded; HUGO, Human Genome Organization; JBI, Joanna Briggs Institute; LEP; lymphoepithelial carcinoma; LR-, Negative Likelihood Ratio; LR+, Positive Likelihood Ratio; MAD, monomorphic adenoma; MEC, mucoepidermoid carcinoma; MSGTs, malignant salivary gland tumors, MYOC, myoepithelial carcinoma; MYO, myoepithelioma; NEC, neuroendocrine carcinoma; NOS, not otherwise specified adenocarcinoma; OncoCa, oncocytic carcinoma; PA, pleomorphic adenoma; PICO, Population, Intervention, Comparator, and Outcome; P(LG)A, polymorphous low-grade adenocarcinoma; poorly differentiated Ca, poorly differentiated carcinoma; PRISMA, Preferred Reporting Items for Systematic Reviews and Meta-Analyses; RoB, risk of bias; SCC, squamous cell carcinoma; SDC, salivary duct carcinoma; SGs, salivary glands; SGTs, salivary gland tumors; SROC, Summary Receiver Operating Characteristic; Undif.Ca, undifferentiated carcinoma, WHO, World Health Organization; WT, Warthin’s tumor
