# Supplementary material for: The Immunoexpression and Prognostic Significance of Stem Cell Markers in Malignant Salivary Gland Tumors: A Systematic Review and Meta-Analysis
Source: Genes (Basel). 2024 Dec 29;16(1):37. doi: 10.3390/genes16010037 (PMC11764928; doi:10.3390/genes16010037)
Supplement: Supplementary file 1 [file genes-16-00037-s001.zip › genes-3374333_Supplementary figures-Revised/Supplementary_Figure_6_KIT_AdCC_MYOC_No_DOR.pdf]

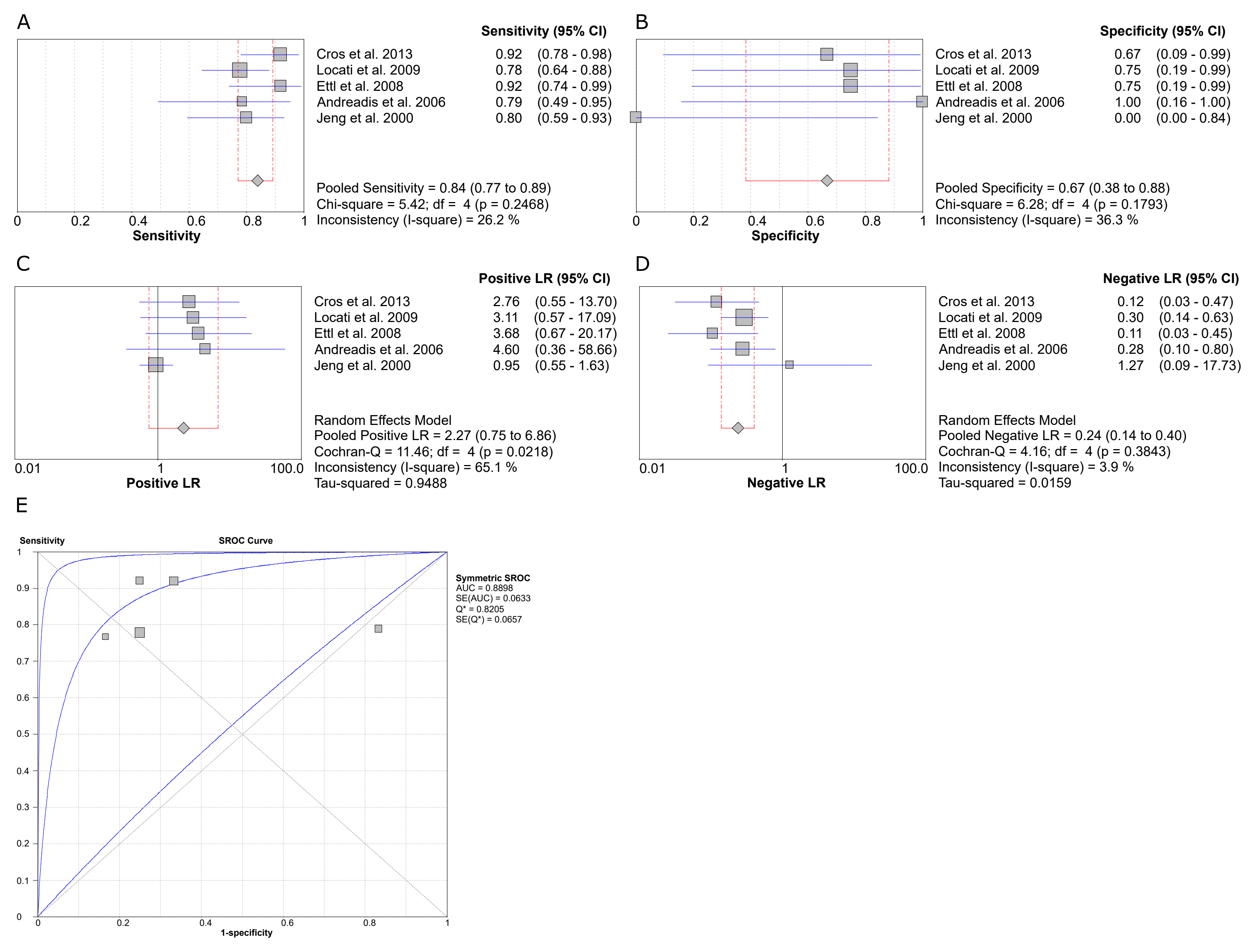
A
Sensitivity (95% CI)
Cros et al. 2013 0.92 (0.78 - 0.98)
Locati et al. 2009 0.78 (0.64 - 0.88)
Ettl et al. 2008 0.92 (0.74 - 0.99)
Andreadis et al. 2006 0.79 (0.49 - 0.95)
Jeng et al. 2000 0.80 (0.59 - 0.93)
Pooled Sensitivity = 0.84 (0.77 to 0.89)
Chi-square = 5.42; df = 4 (p = 0.2468)
Inconsistency (I-square) = 26.2 %
0
0.2
0.4
0.6
0.8
1
Sensitivity
B
Specificity (95% CI)
Cros et al. 2013 0.67 (0.09 - 0.99)
Locati et al. 2009 0.75 (0.19 - 0.99)
Ettl et al. 2008 0.75 (0.19 - 0.99)
Andreadis et al. 2006 1.00 (0.16 - 1.00)
Jeng et al. 2000 0.00 (0.00 - 0.84)
Pooled Specificity = 0.67 (0.38 to 0.88)
Chi-square = 6.28; df = 4 (p = 0.1793)
Inconsistency (I-square) = 36.3 %
0
0.2
0.4
0.6
0.8
1
Specificity
C
Positive LR (95% CI)
Cros et al. 2013 2.76 (0.55 - 13.70)
Locati et al. 2009 3.11 (0.57 - 17.09)
Ettl et al. 2008 3.68 (0.67 - 20.17)
Andreadis et al. 2006 4.60 (0.36 - 58.66)
Jeng et al. 2000 0.95 (0.55 - 1.63)
Random Effects Model
Pooled Positive LR = 2.27 (0.75 to 6.86)
Cochran-Q = 11.46; df = 4 (p = 0.0218)
Inconsistency (I-square) = 65.1 %
Tau-squared = 0.9488
0.01
1
100.0
Positive LR
D
Negative LR (95% CI)
Cros et al. 2013 0.12 (0.03 - 0.47)
Locati et al. 2009 0.30 (0.14 - 0.63)
Ettl et al. 2008 0.11 (0.03 - 0.45)
Andreadis et al. 2006 0.28 (0.10 - 0.80)
Jeng et al. 2000 1.27 (0.09 - 17.73)
Random Effects Model
Pooled Negative LR = 0.24 (0.14 to 0.40)
Cochran-Q = 4.16; df = 4 (p = 0.3843)
Inconsistency (I-square) = 3.9 %
Tau-squared = 0.0159
0.01
1
100.0
Negative LR
E
Sensitivity
SROC Curve
1
0.9
0.8
0.7
0.6
0.5
0.4
0.3
0.2
0.1
0
0
0.2
0.4
0.6
0.8
1
1-specificity
Symmetric SROC
AUC = 0.8898
SE(AUC) = 0.0633
Q* = 0.8205
SE(Q*) = 0.0657
