# Supplementary figures and images for: The Immunoexpression and Prognostic Significance of Stem Cell Markers in Malignant Salivary Gland Tumors: A Systematic Review and Meta-Analysis
Source: Genes (Basel). 2024 Dec 29;16(1):37. doi: 10.3390/genes16010037 (PMC11764928; doi:10.3390/genes16010037)

A

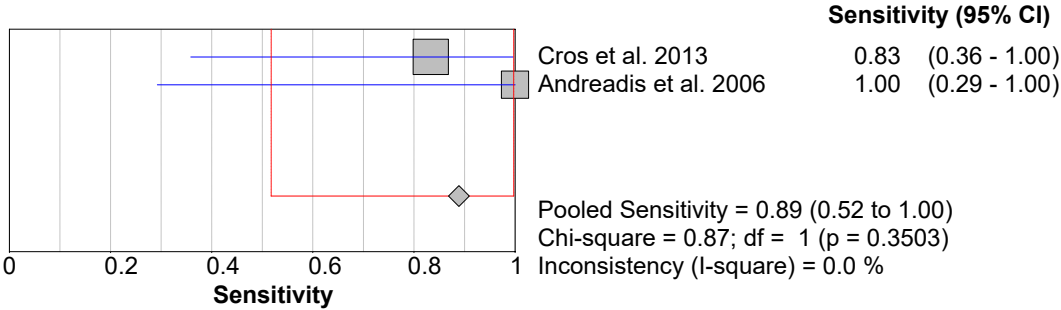

B

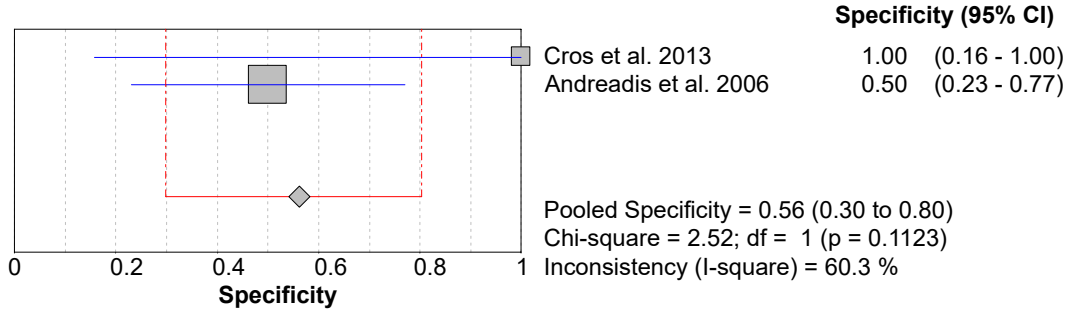

C

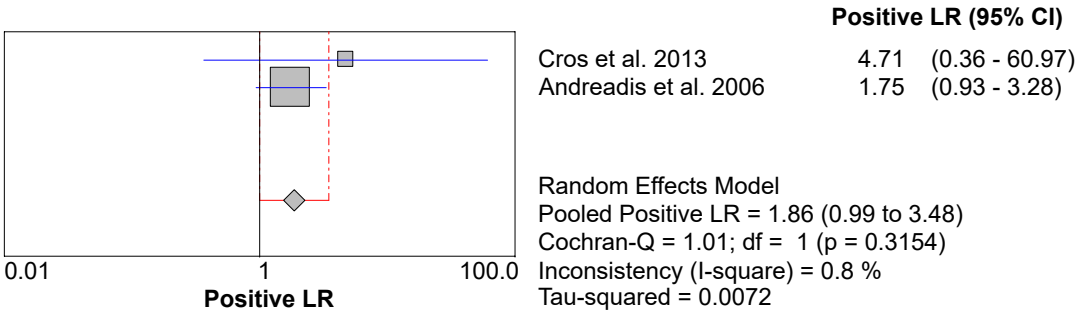

D

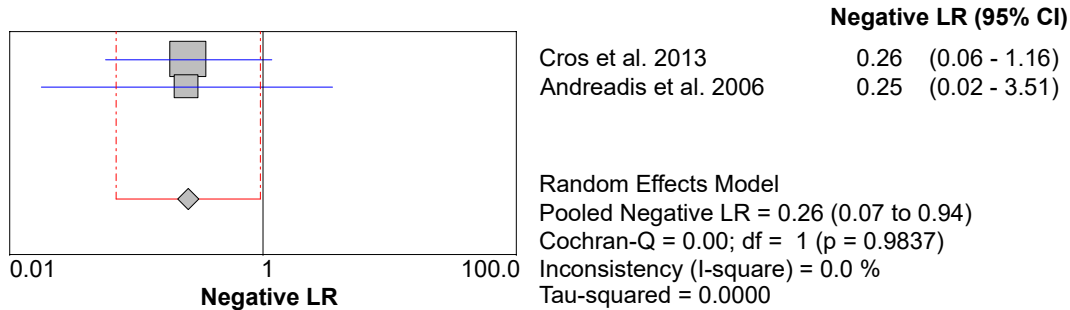

E

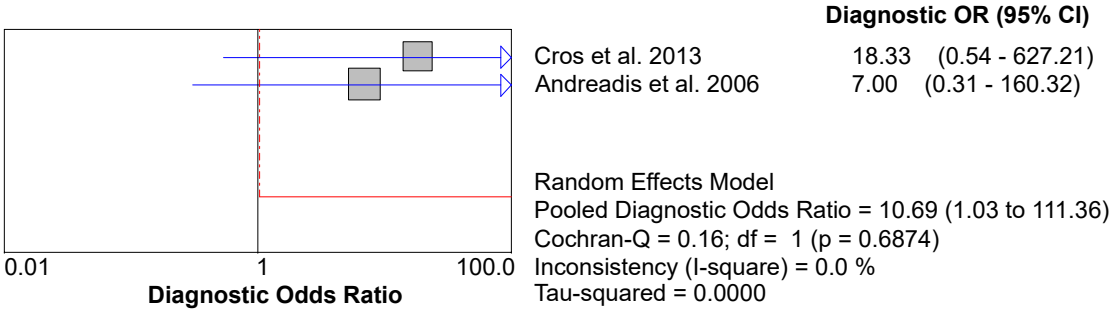

F

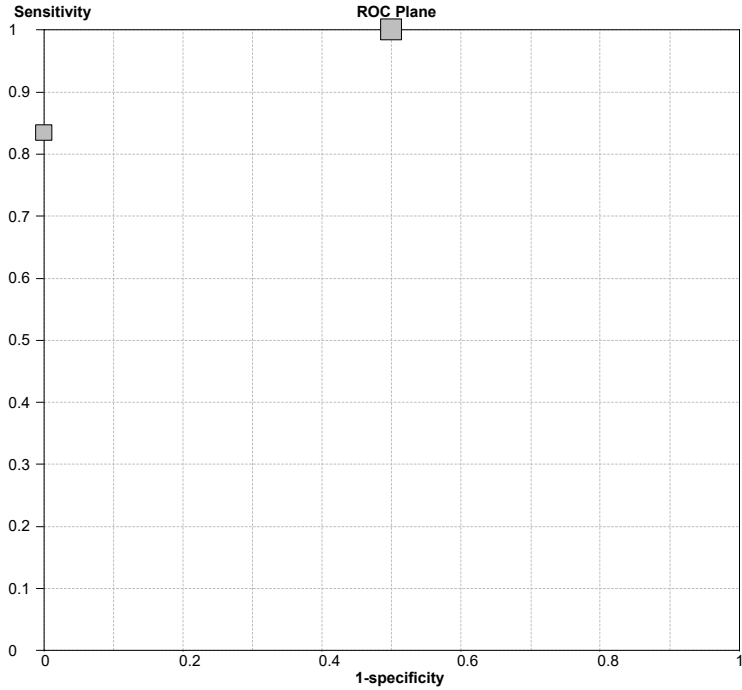

Supplement: Supplementary file 1 [file genes-16-00037-s001.zip › genes-3374333_Supplementary figures-Revised/Supplementary_Figure_10_KIT_EMC_PLGA.pdf]

A

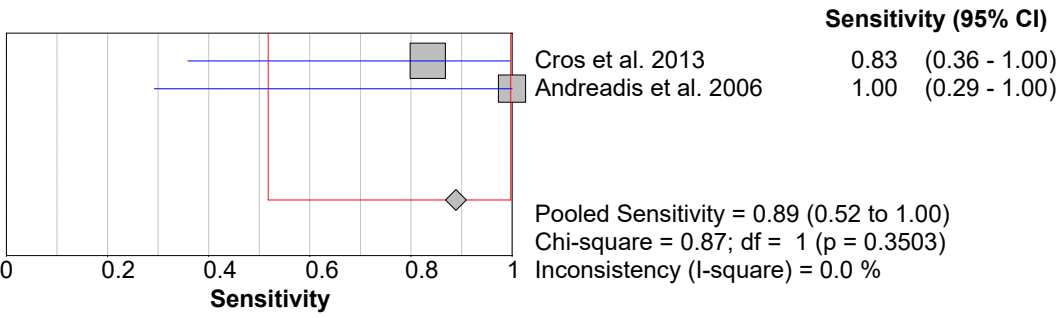

B

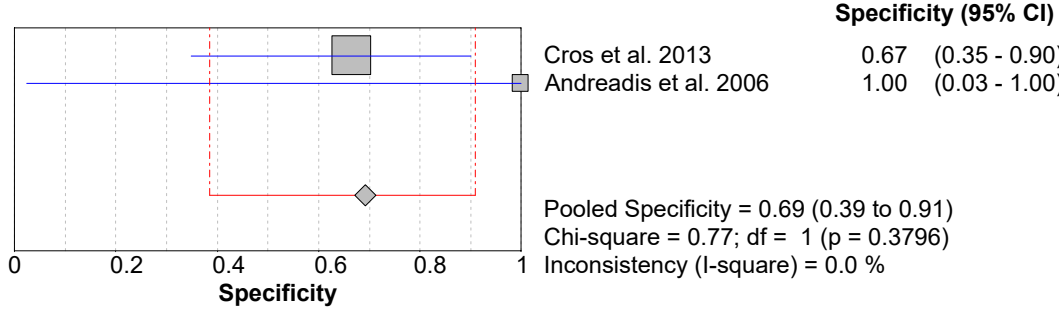

C

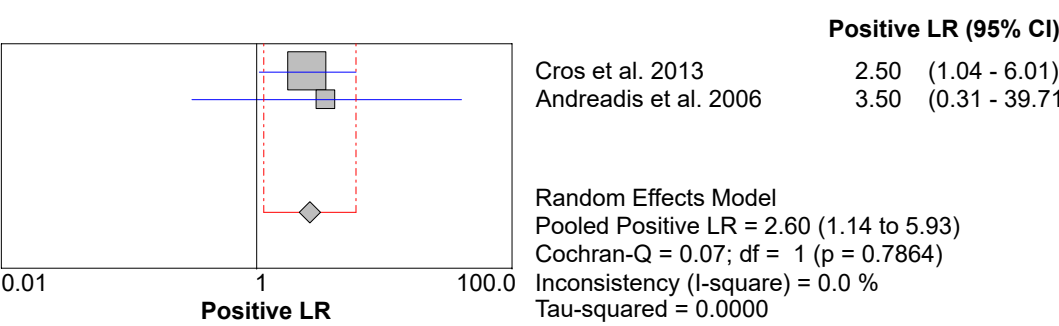

D

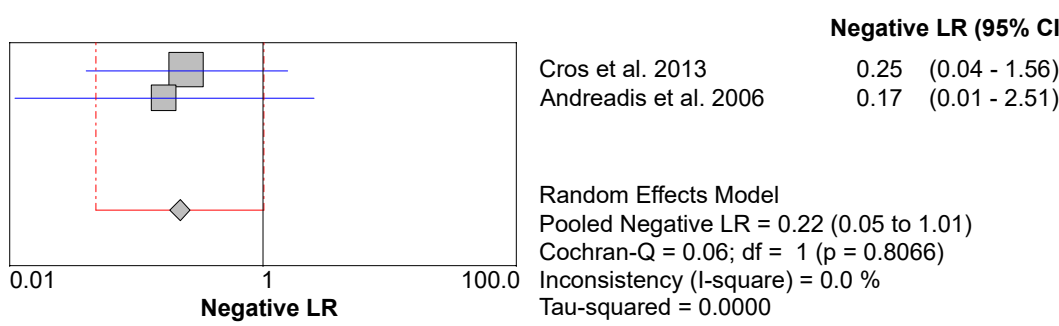

E

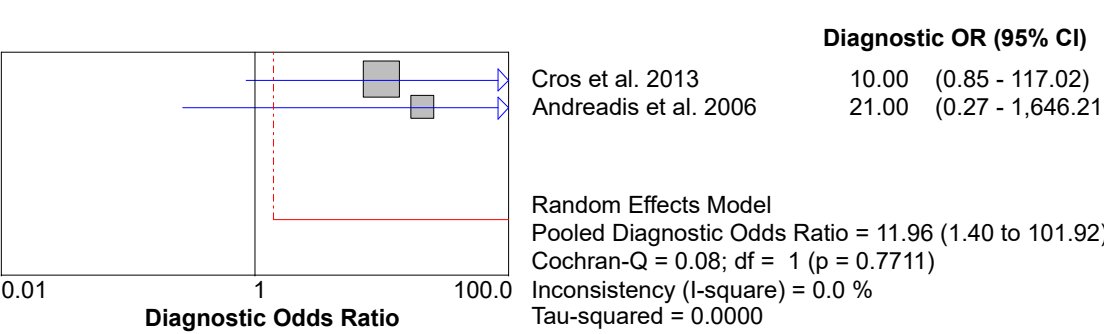

F

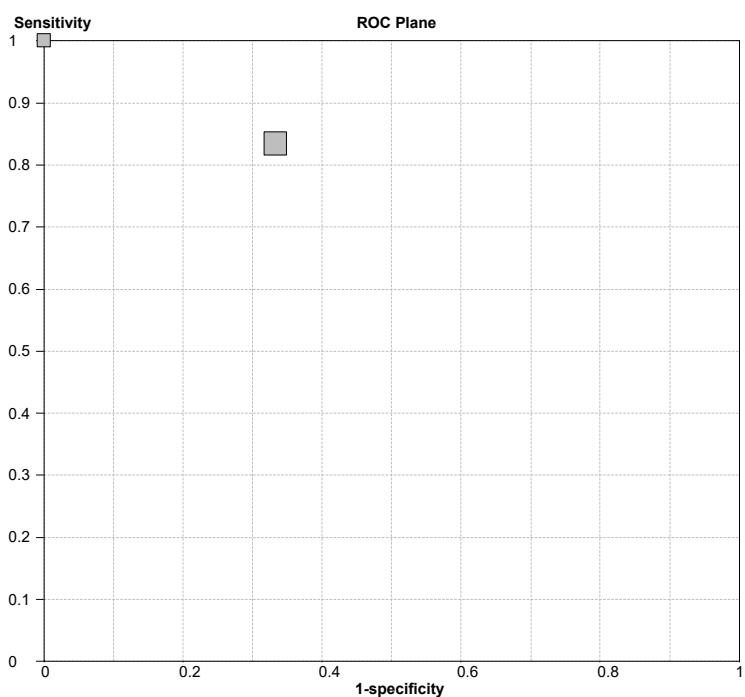

Supplement: Supplementary file 1 [file genes-16-00037-s001.zip › genes-3374333_Supplementary figures-Revised/Supplementary_Figure_11_KIT_EMC_NOS.pdf]

A

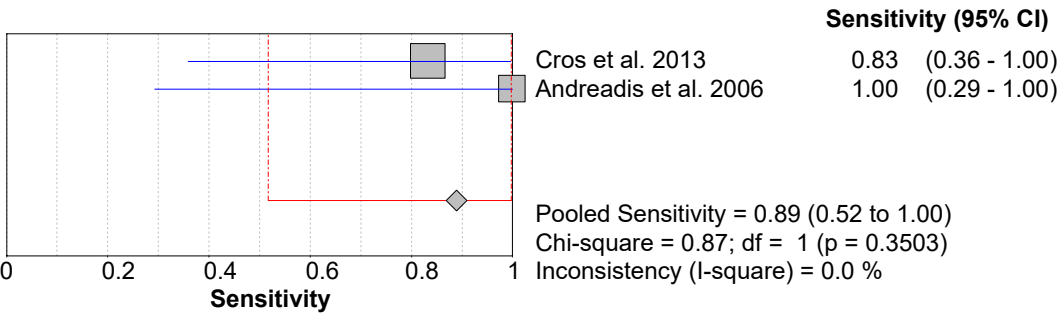

B

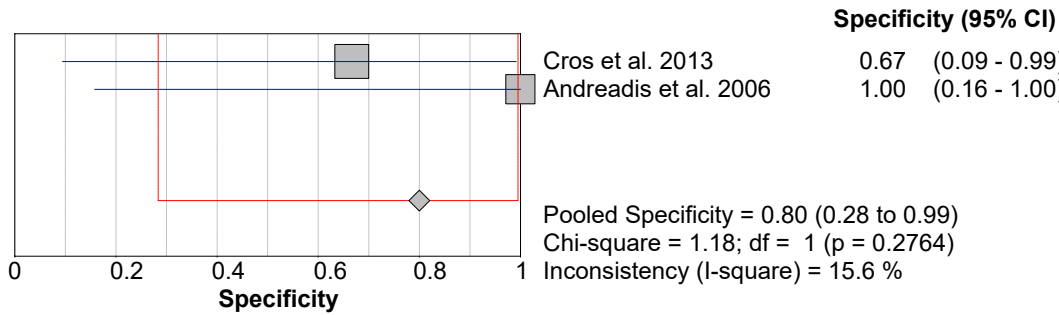

C

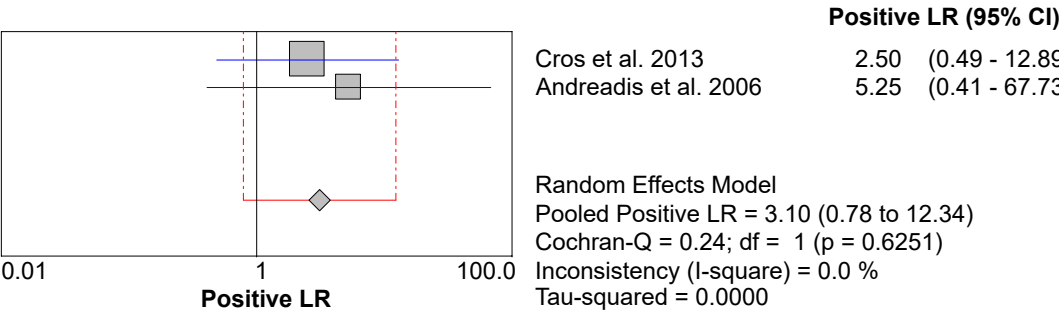

D

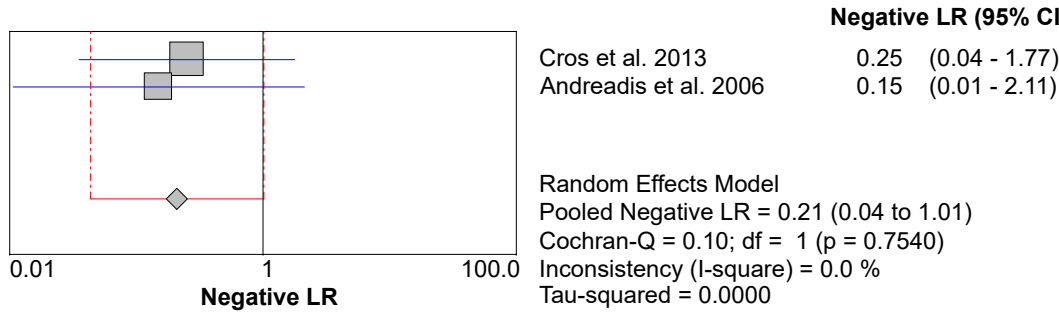

E

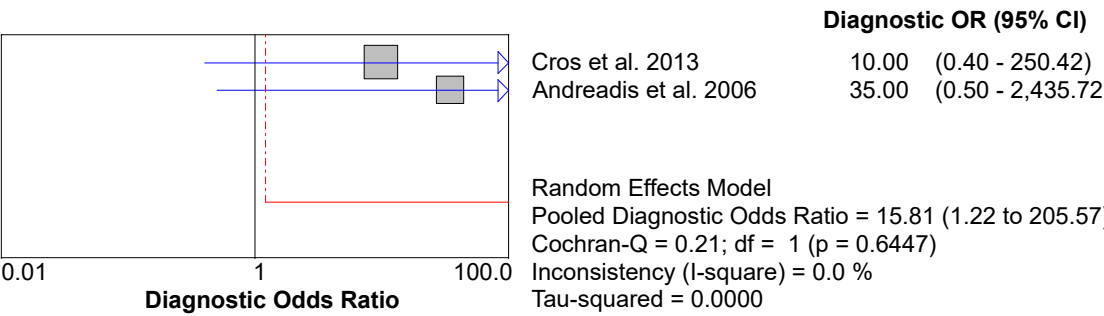

F

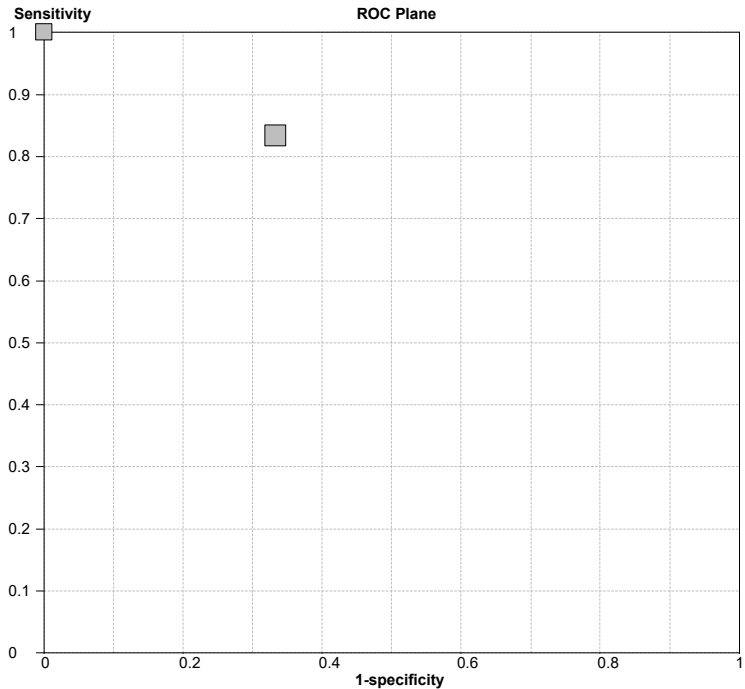

Supplement: Supplementary file 1 [file genes-16-00037-s001.zip › genes-3374333_Supplementary figures-Revised/Supplementary_Figure_12_KIT_EMC_MYOC.pdf]

A

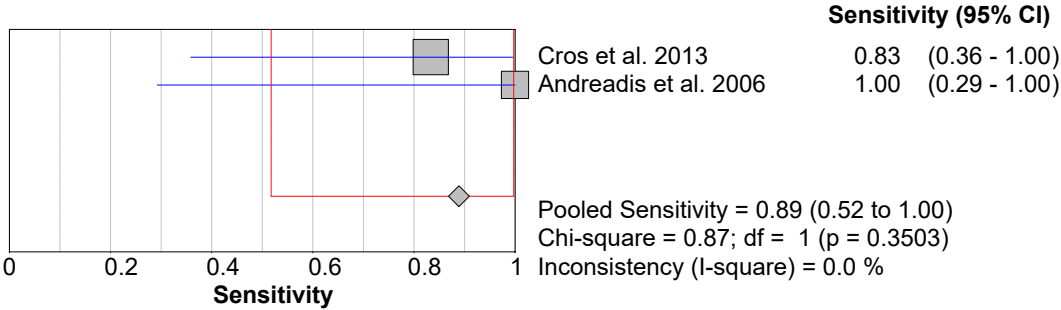

B

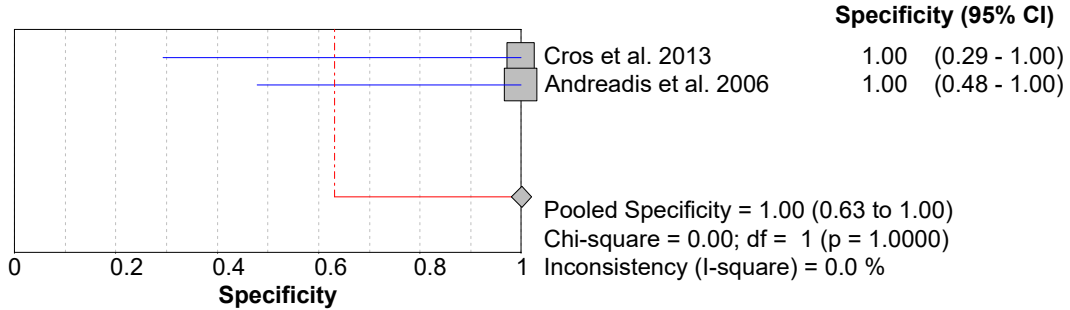

C

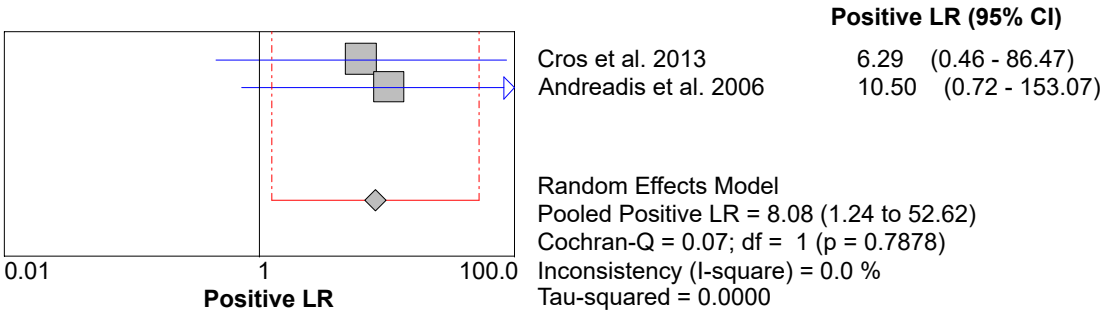

D

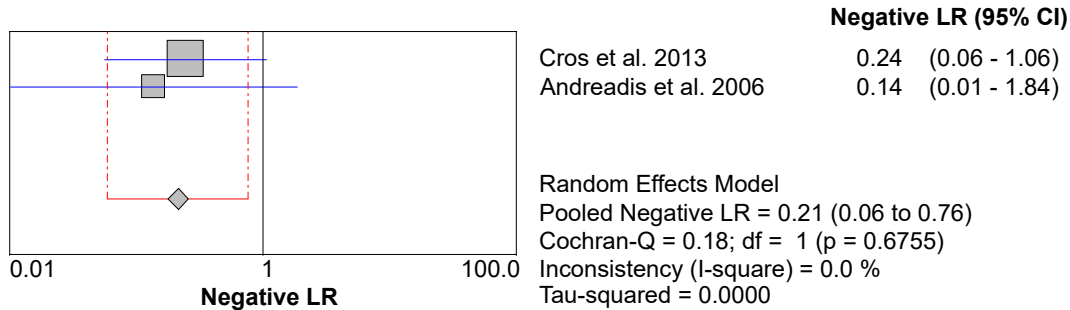

E

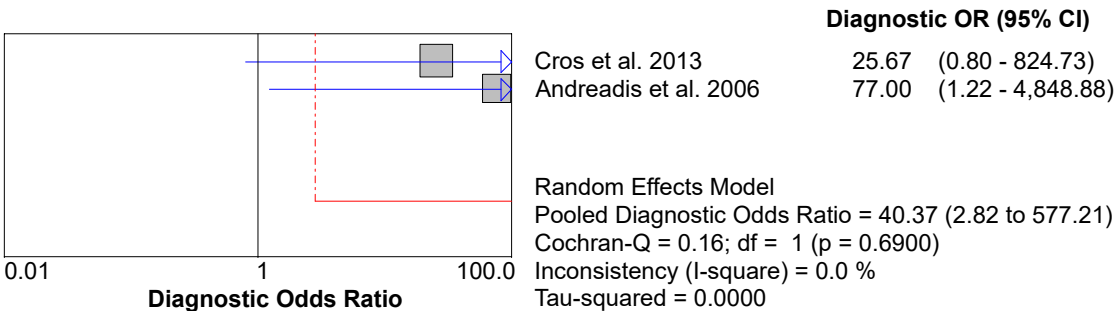

F

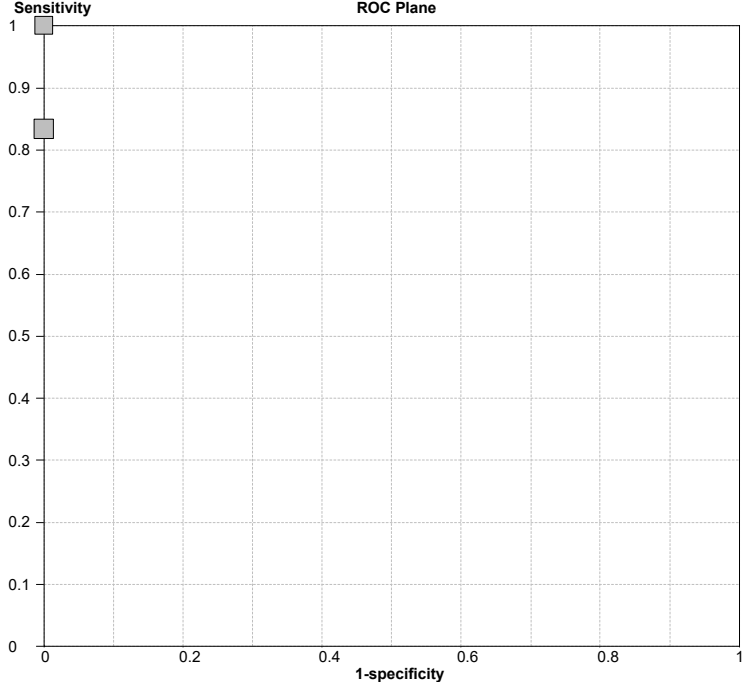

Supplement: Supplementary file 1 [file genes-16-00037-s001.zip › genes-3374333_Supplementary figures-Revised/Supplementary_Figure_13_KIT_EMC_SDC.pdf]

A

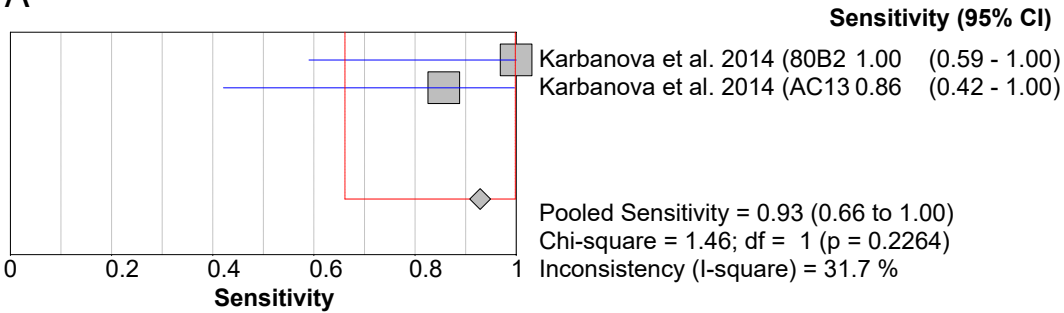

B

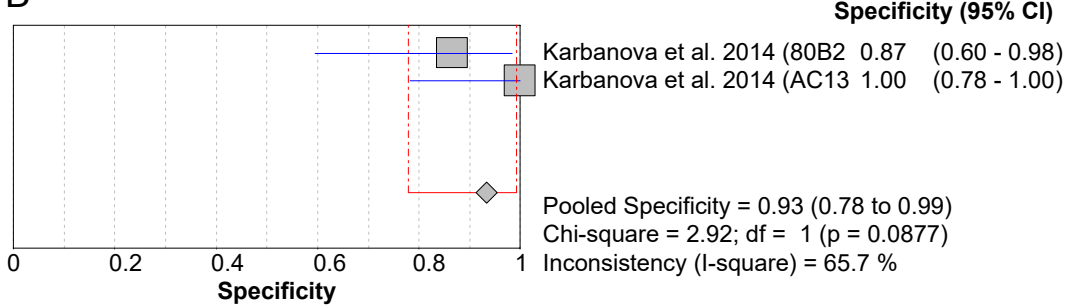

C

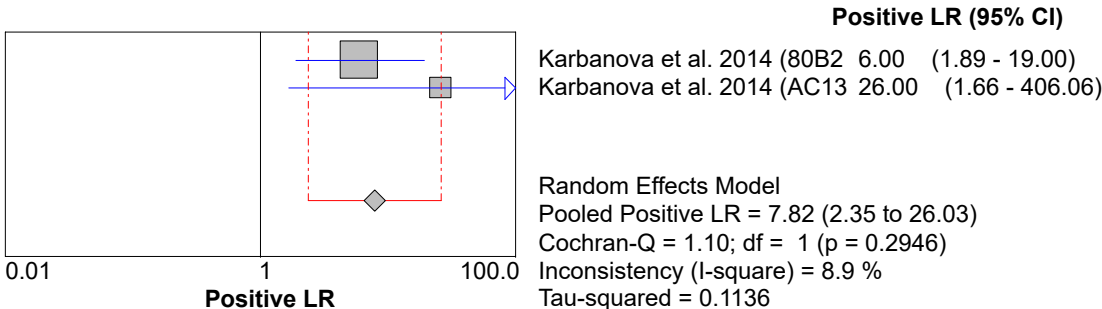

D

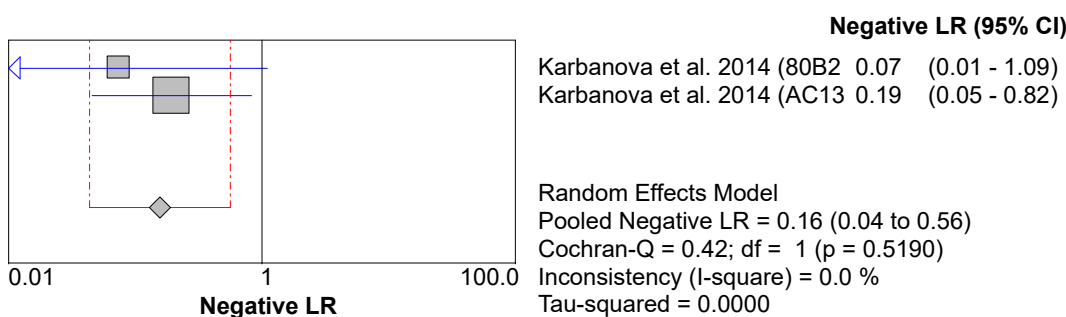

E

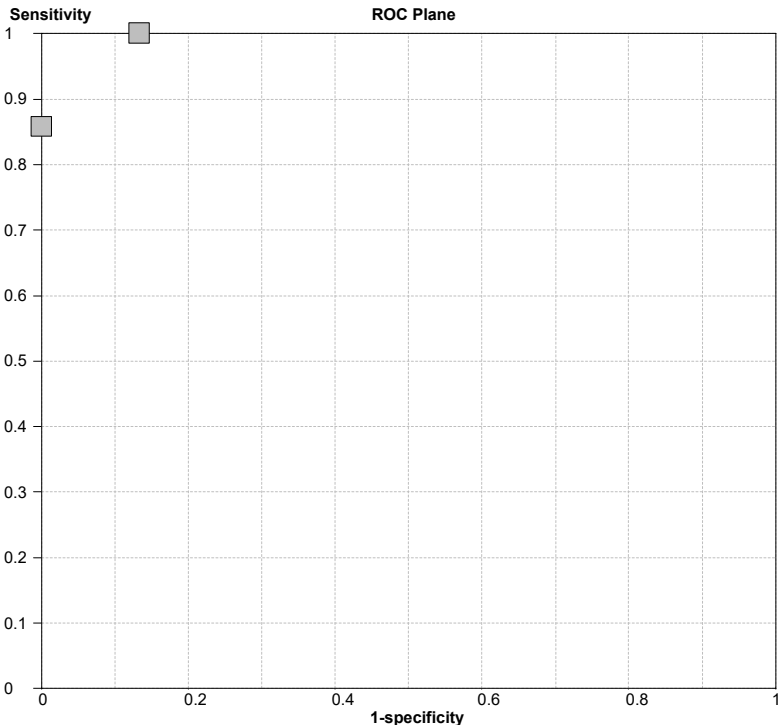

Supplement: Supplementary file 1 [file genes-16-00037-s001.zip › genes-3374333_Supplementary figures-Revised/Supplementary_Figure_14_CD133_AdCC_MEC_No_DOR.pdf]

A

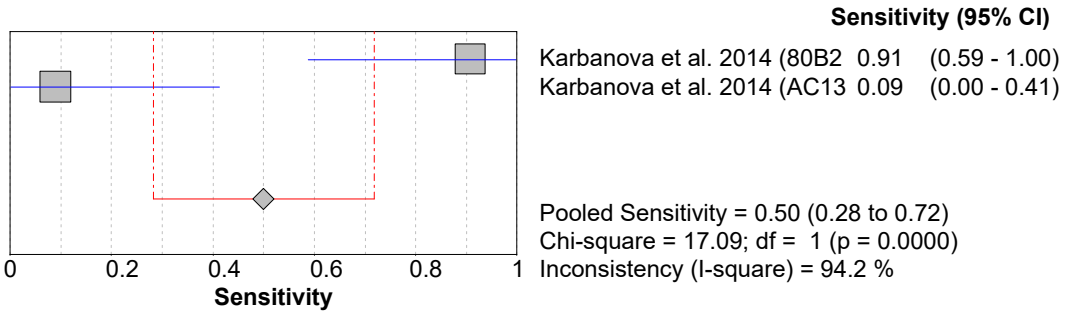

B

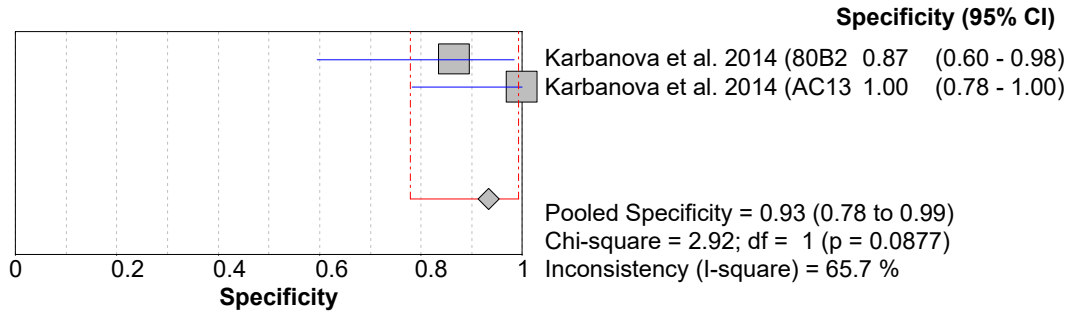

C

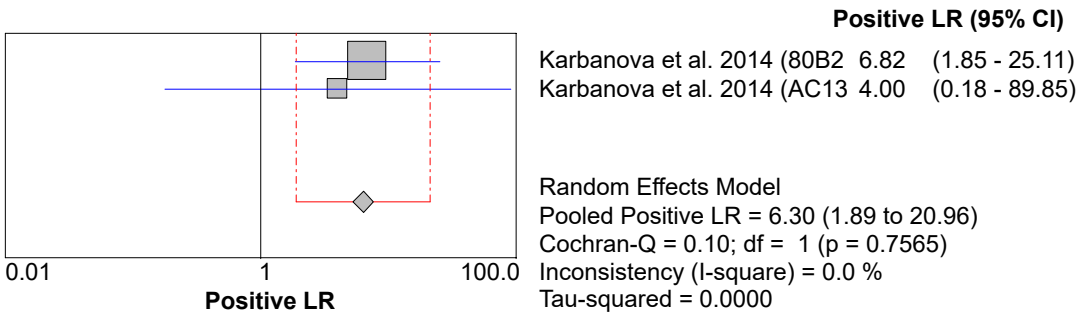

D

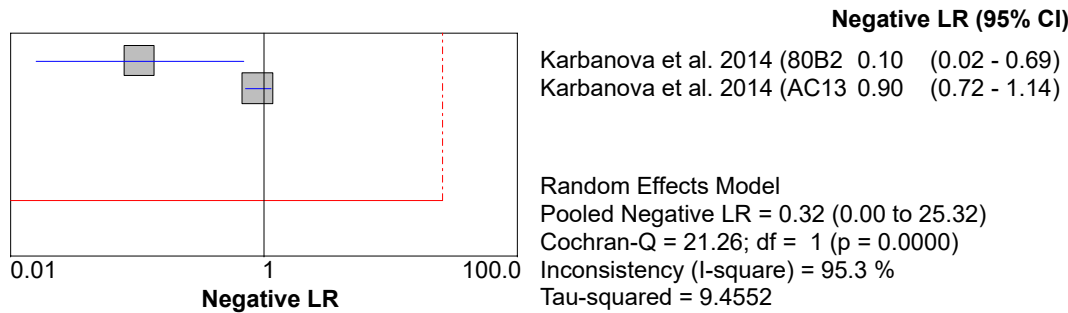

E

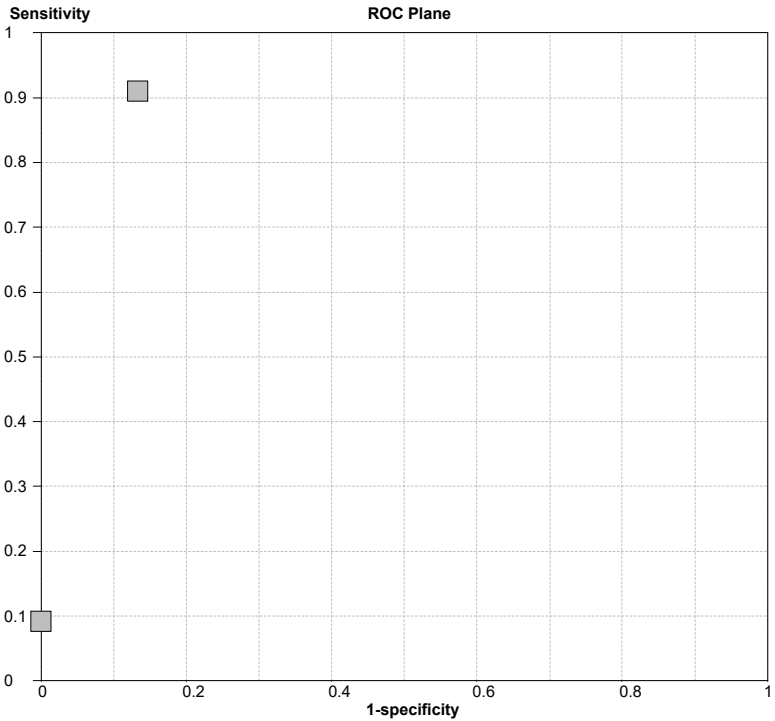

Supplement: Supplementary file 1 [file genes-16-00037-s001.zip › genes-3374333_Supplementary figures-Revised/Supplementary_Figure_15_CD133_ACC_MEC_No_DOR.pdf]

A

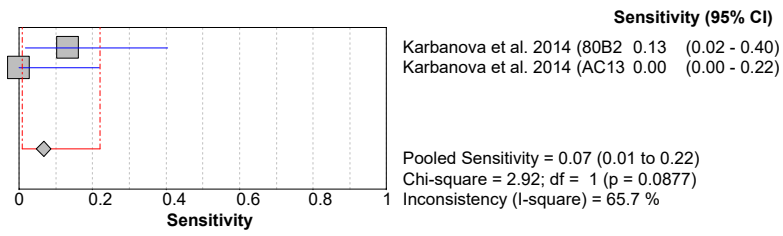

B

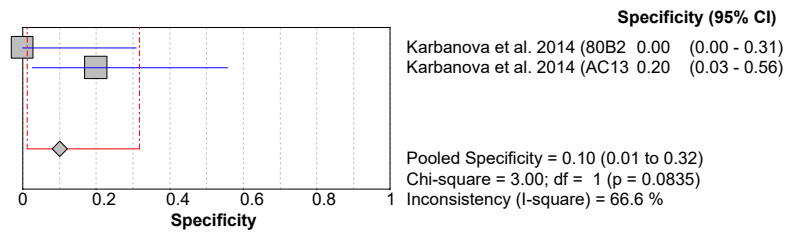

C

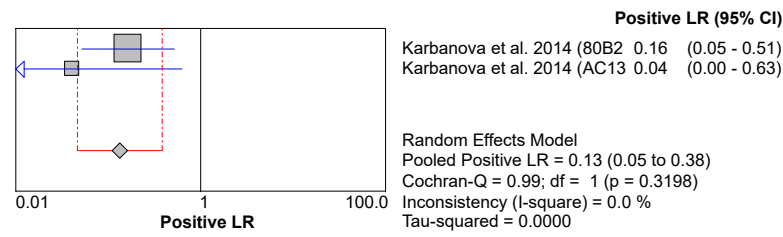

D

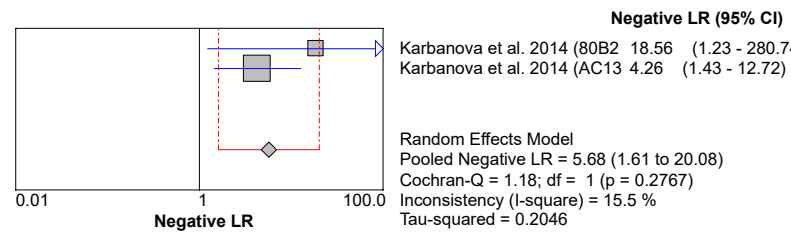

E

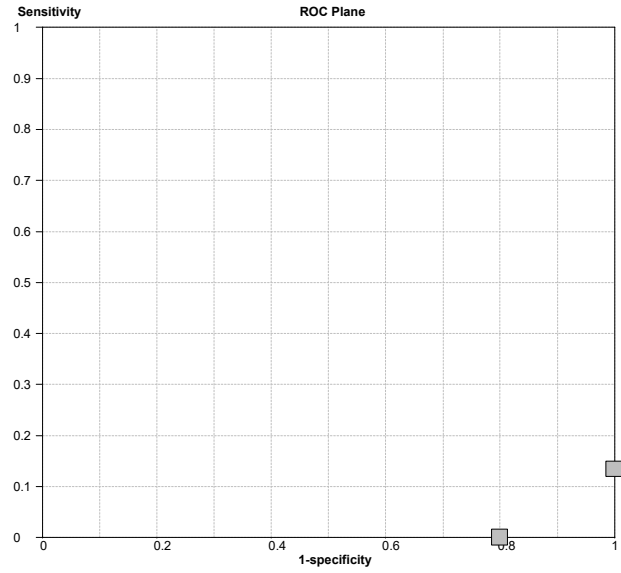

Supplement: Supplementary file 1 [file genes-16-00037-s001.zip › genes-3374333_Supplementary figures-Revised/Supplementary_Figure_16_CD133_MEC_PA_No_DOR.pdf]

A

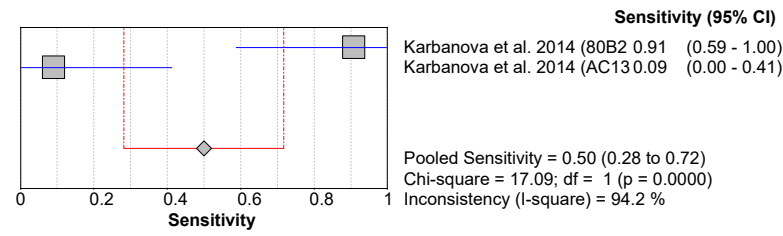

B

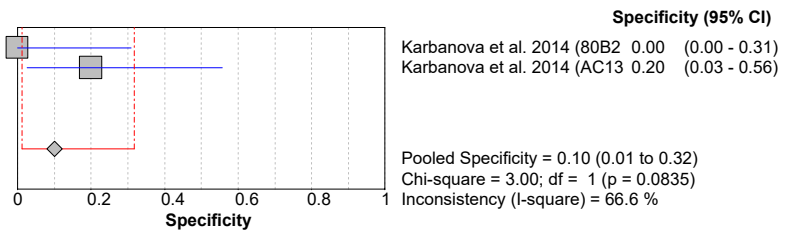

C

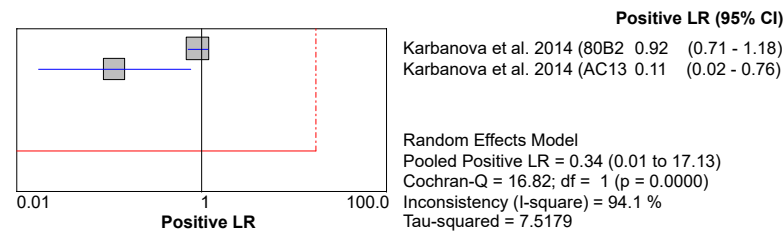

D

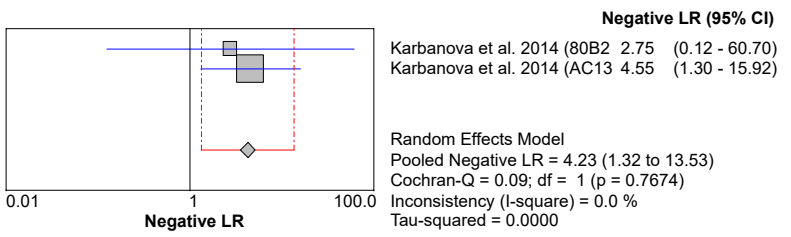

E

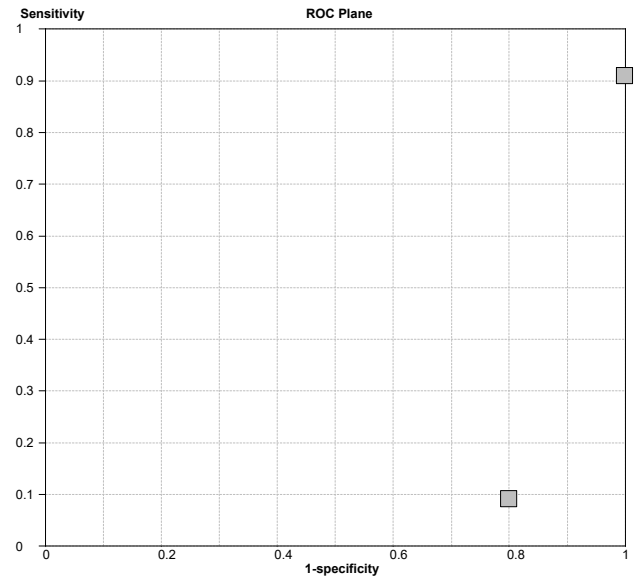

Supplement: Supplementary file 1 [file genes-16-00037-s001.zip › genes-3374333_Supplementary figures-Revised/Supplementary_Figure_17_CD133_ACC_PA_No_DOR.pdf]

**A**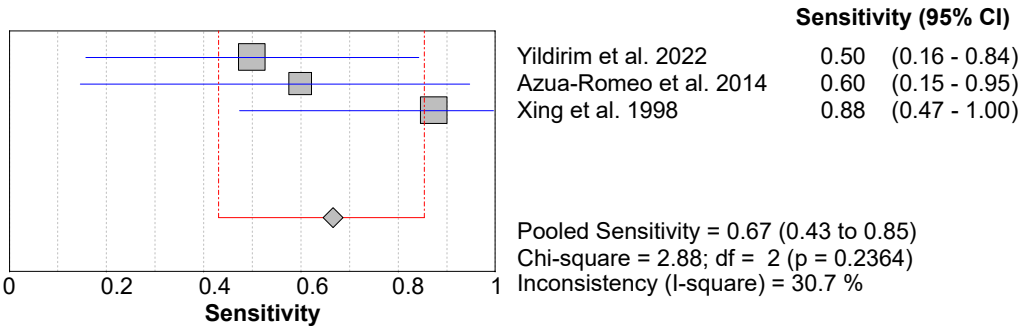**B**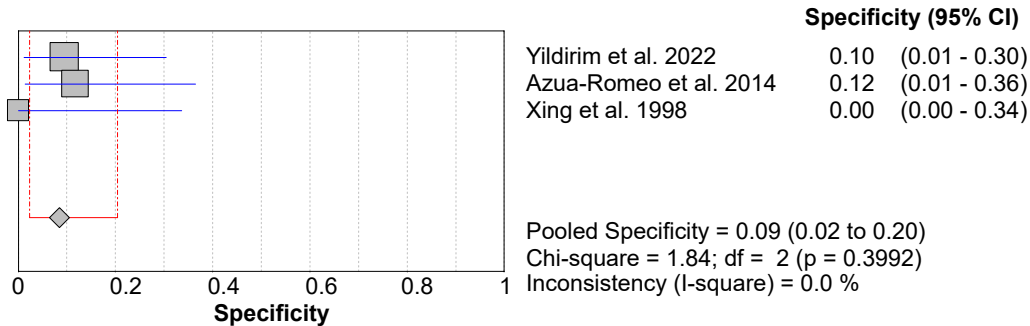**C**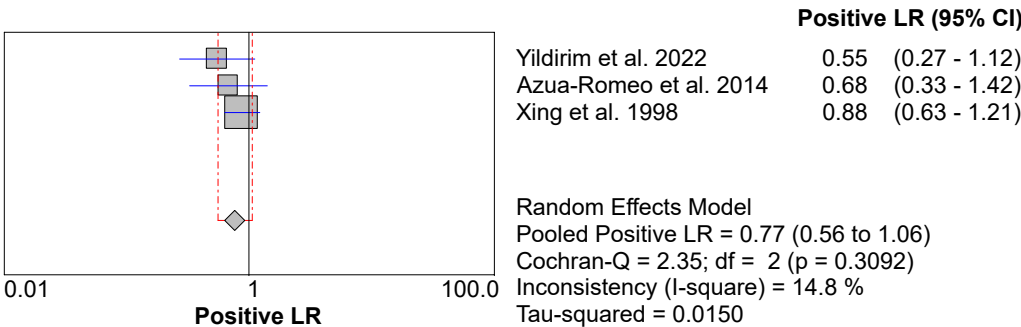**D**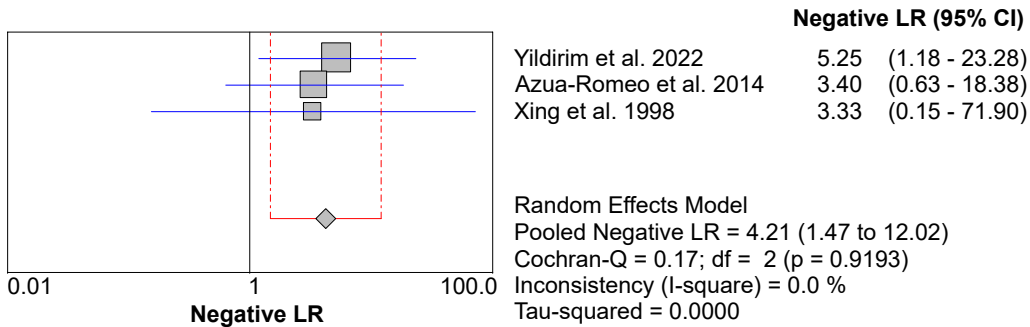**E**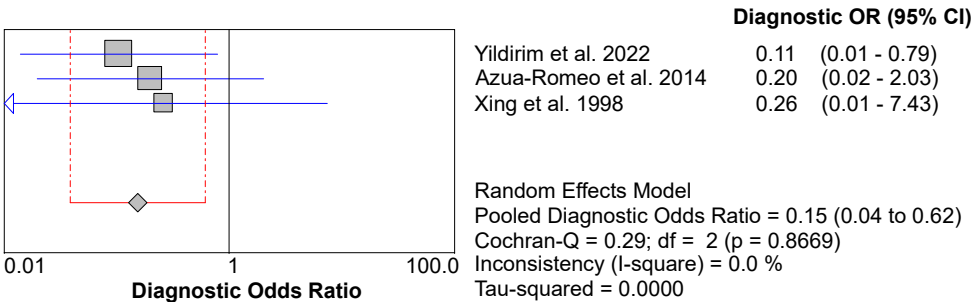**F**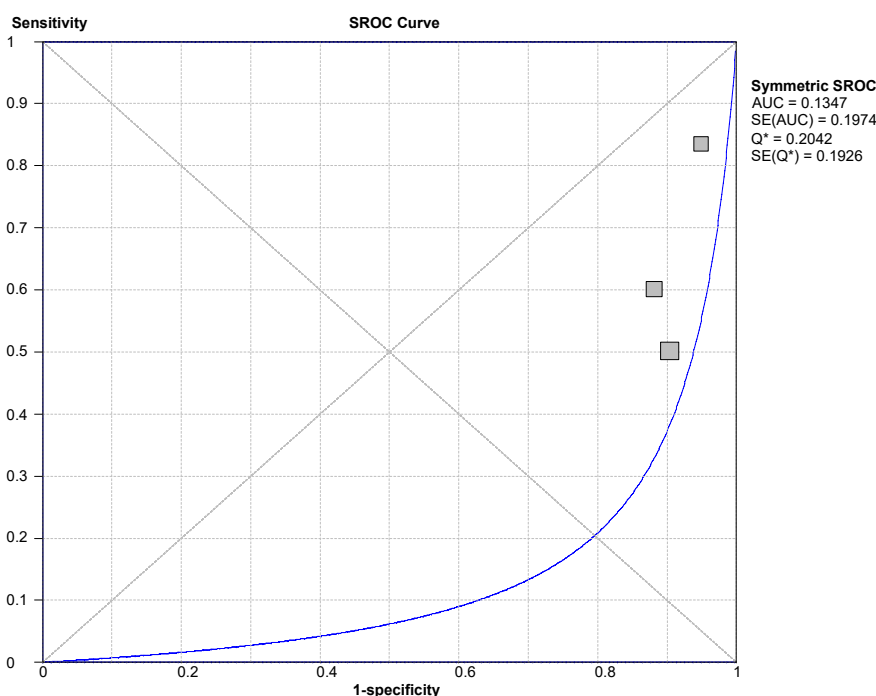

Supplement: Supplementary file 1 [file genes-16-00037-s001.zip › genes-3374333_Supplementary figures-Revised/Supplementary_Figure_18_CD44_AdCC_PA.pdf]

A

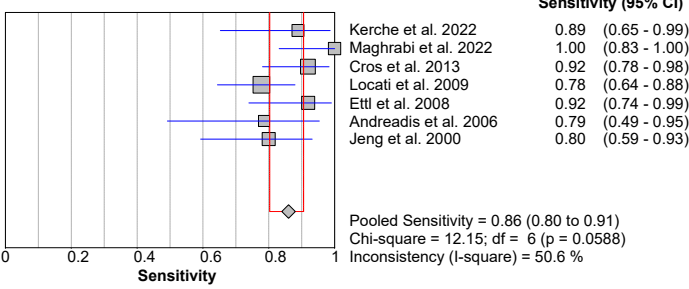

B

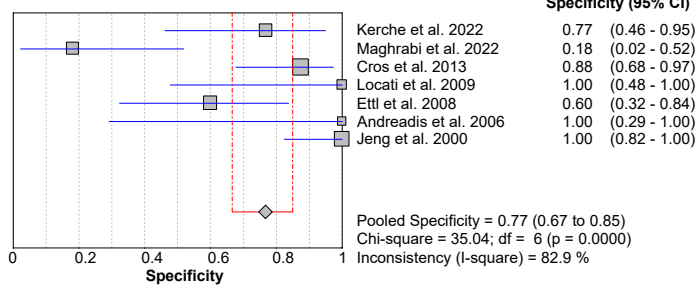

C

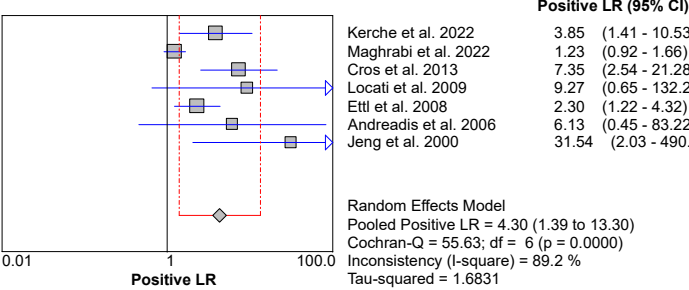

D

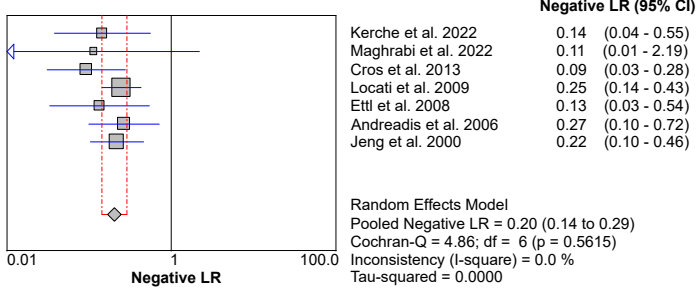

E

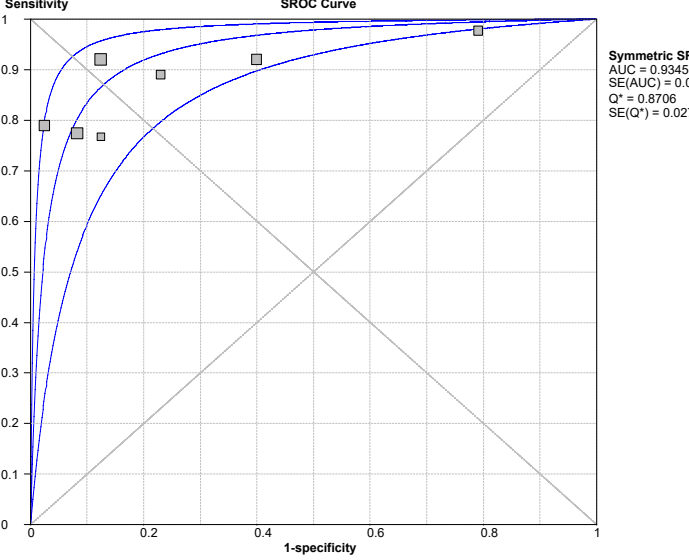

Supplement: Supplementary file 1 [file genes-16-00037-s001.zip › genes-3374333_Supplementary figures-Revised/Supplementary_Figure_1_KIT_AdCC_MEC_No_DOR.pdf]

A

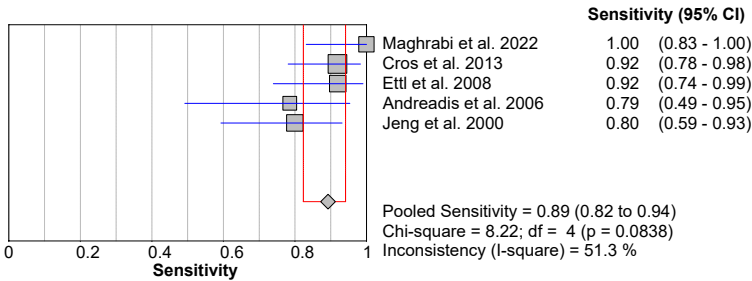

B

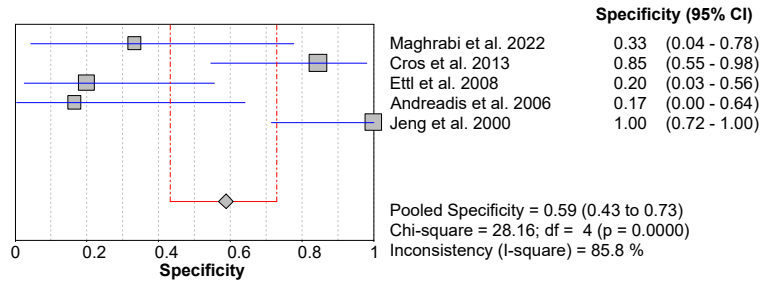

C

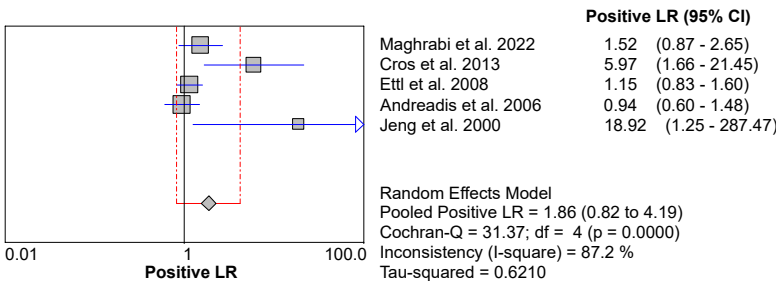

D

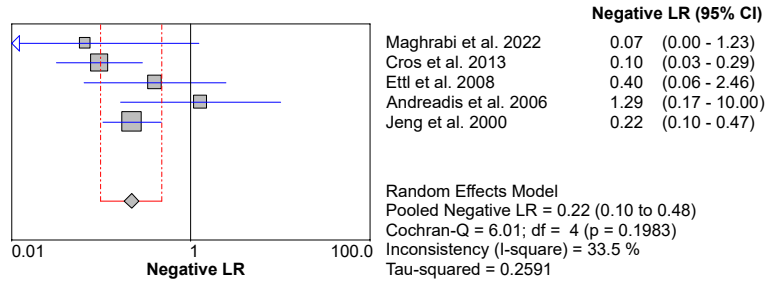

E

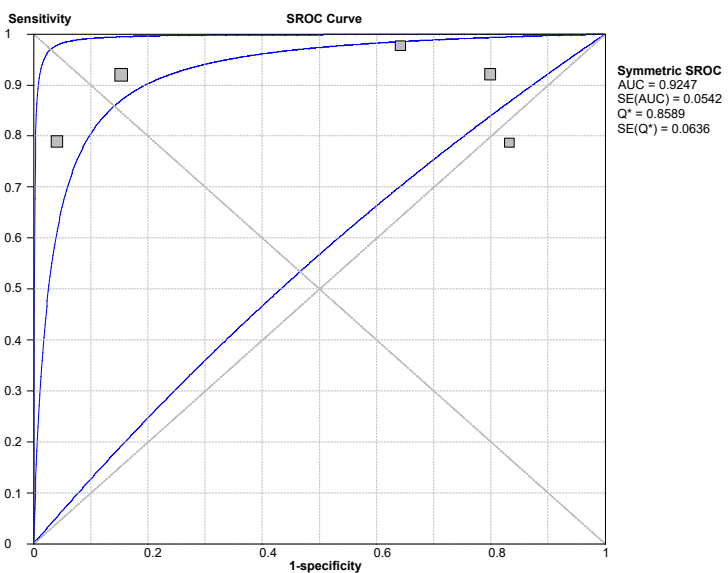

Supplement: Supplementary file 1 [file genes-16-00037-s001.zip › genes-3374333_Supplementary figures-Revised/Supplementary_Figure_2_KIT_AdCC_ACC_No_DOR.pdf]

A

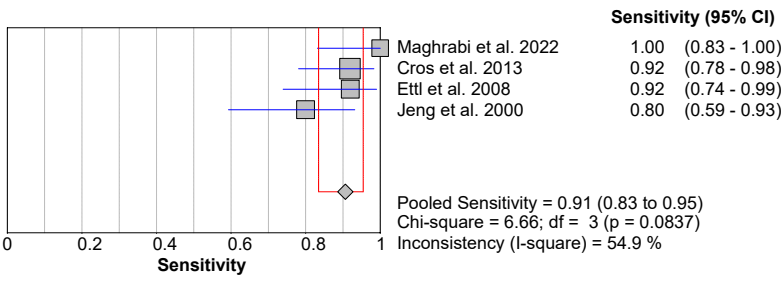

B

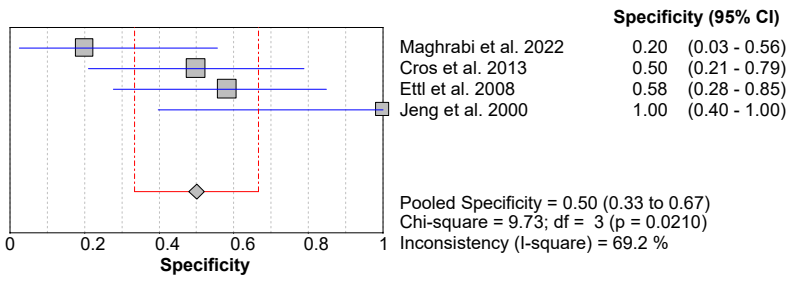

C

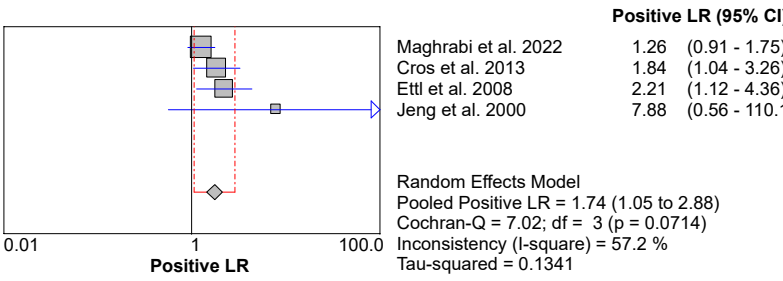

D

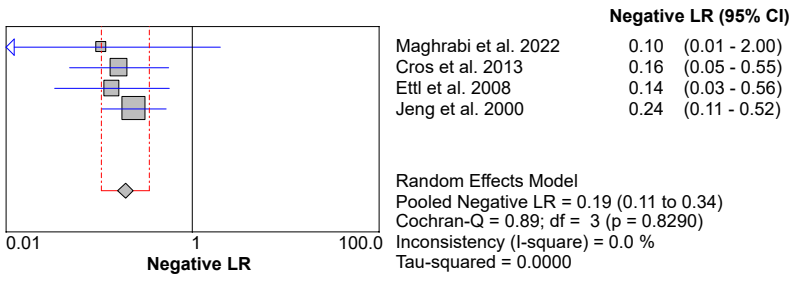

E

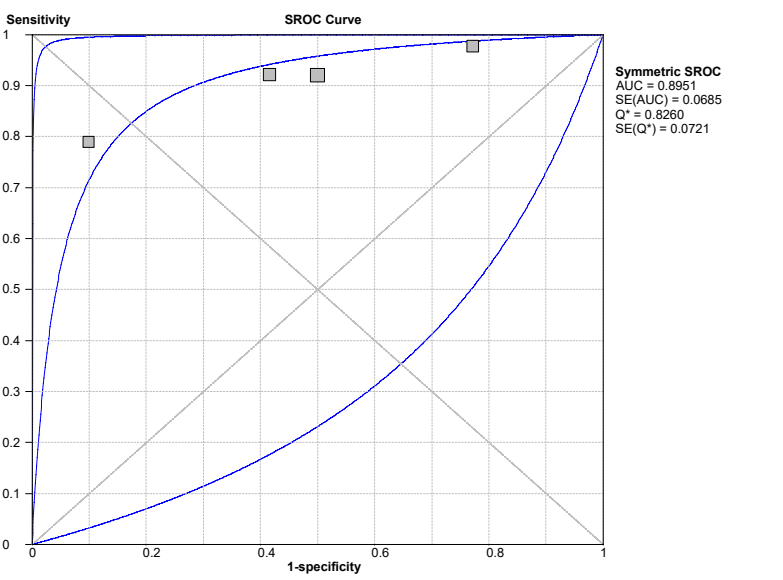

Supplement: Supplementary file 1 [file genes-16-00037-s001.zip › genes-3374333_Supplementary figures-Revised/Supplementary_Figure_3_KIT_AdCC_CXPA_No_DOR.pdf]

A

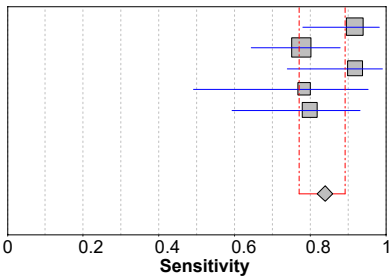

B

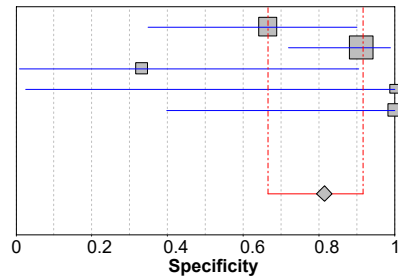

C

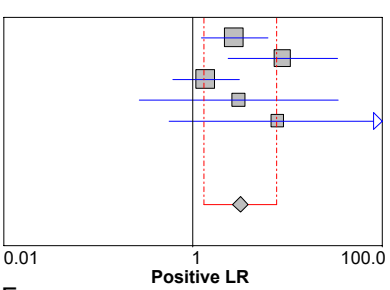

D

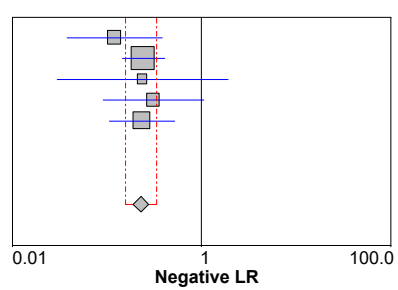

E

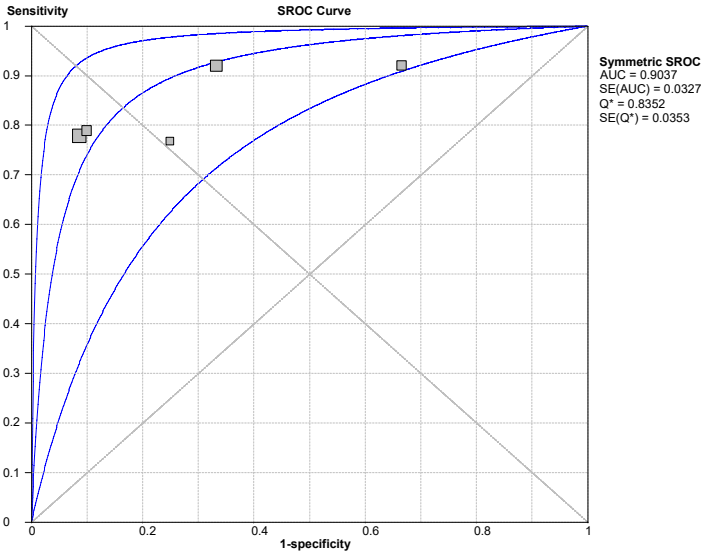

Supplement: Supplementary file 1 [file genes-16-00037-s001.zip › genes-3374333_Supplementary figures-Revised/Supplementary_Figure_4_KIT_AdCC_NOS_No_DOR.pdf]

A

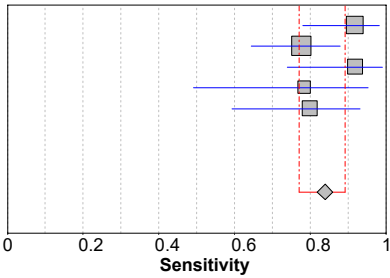

B

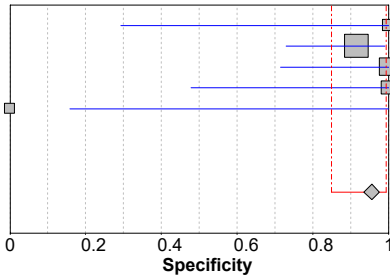

C

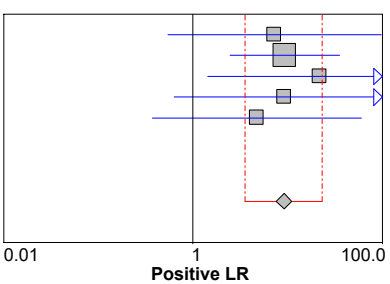

D

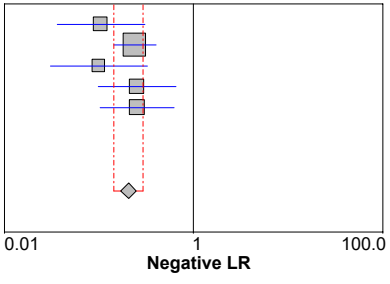

E

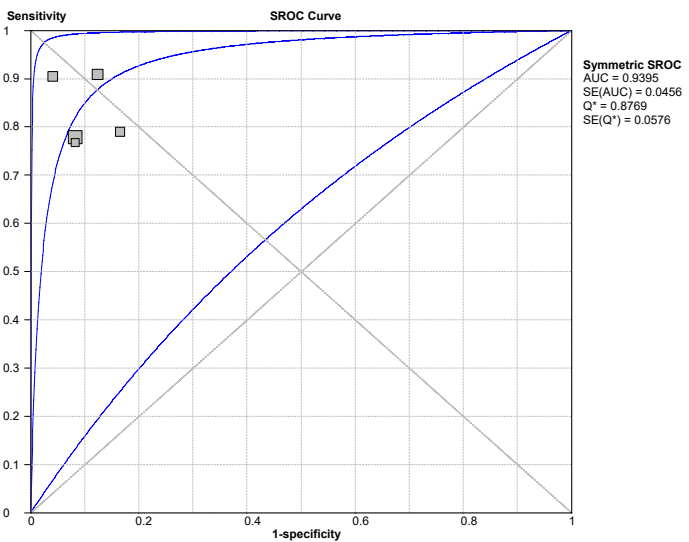

Supplement: Supplementary file 1 [file genes-16-00037-s001.zip › genes-3374333_Supplementary figures-Revised/Supplementary_Figure_5_KIT_AdCC_SDC_No_DOR.pdf]

A

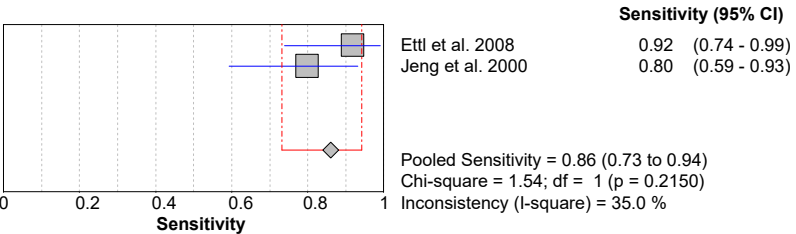

B

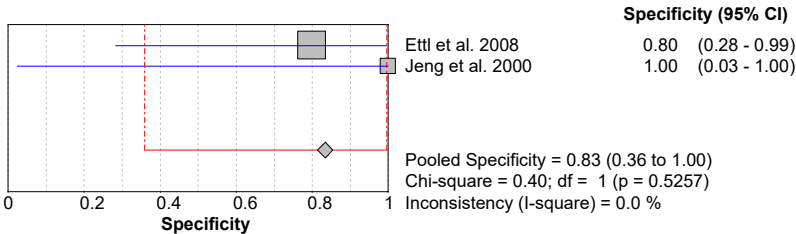

C

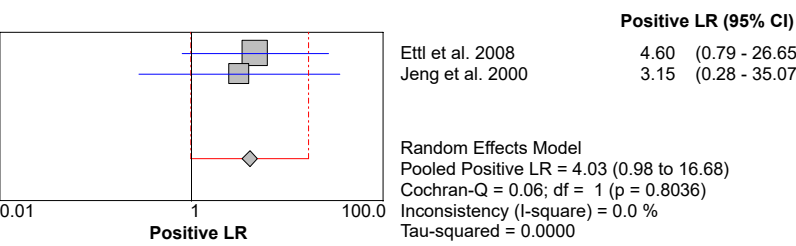

D

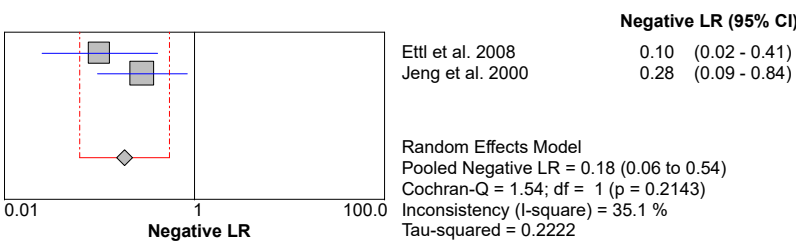

E

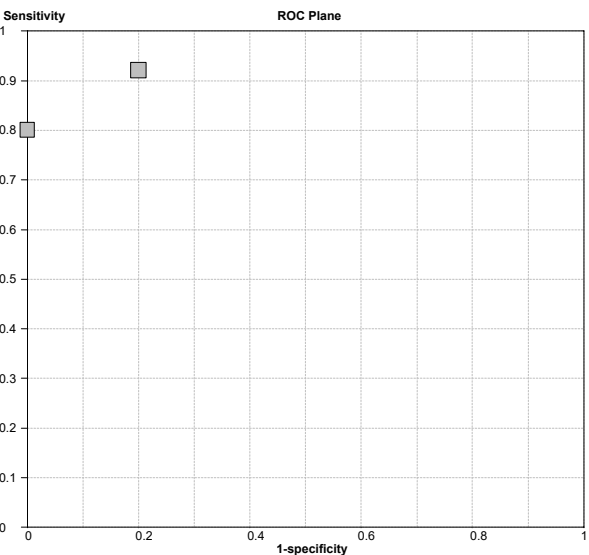

Supplement: Supplementary file 1 [file genes-16-00037-s001.zip › genes-3374333_Supplementary figures-Revised/Supplementary_Figure_7_KIT_AdCC_SCC_No_DOR.pdf]

**A**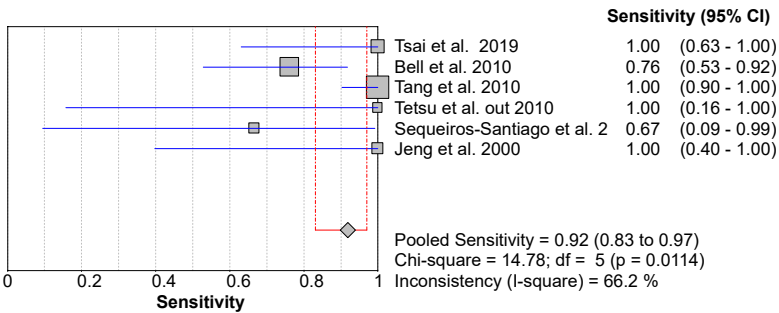**B**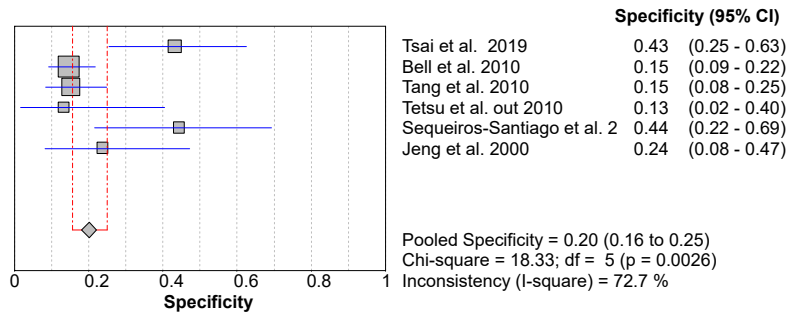**C**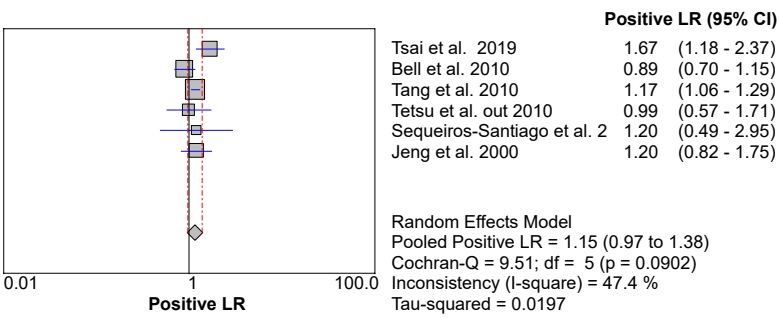**D**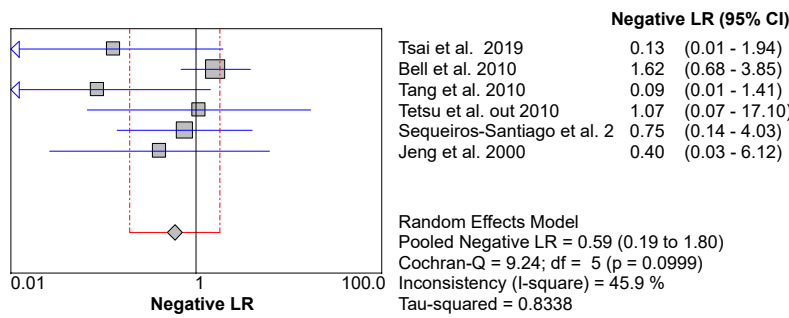**E**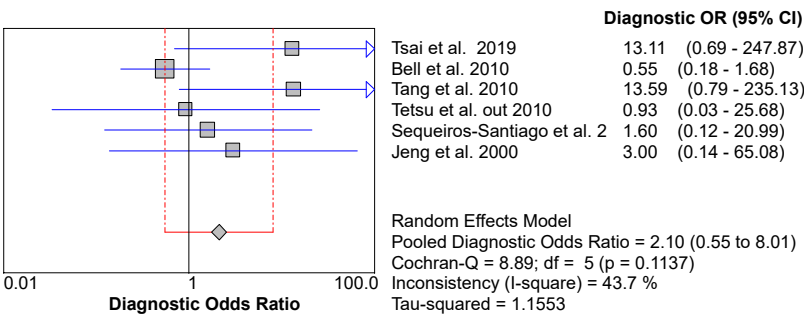**F**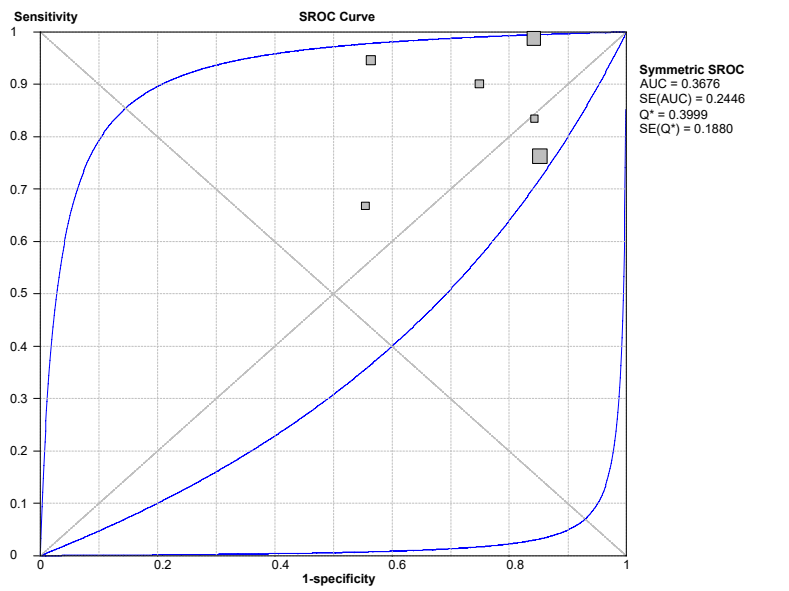

Supplement: Supplementary file 1 [file genes-16-00037-s001.zip › genes-3374333_Supplementary figures-Revised/Supplementary_Figure_8_KIT_solid_cribriform-tubular_AdCC.pdf]

A

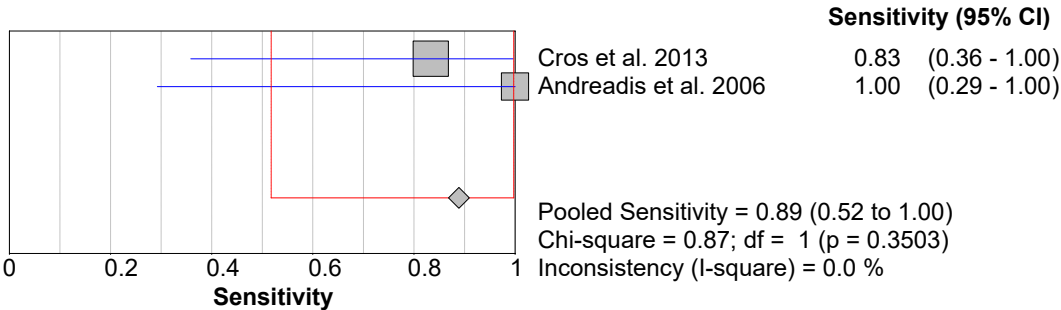

B

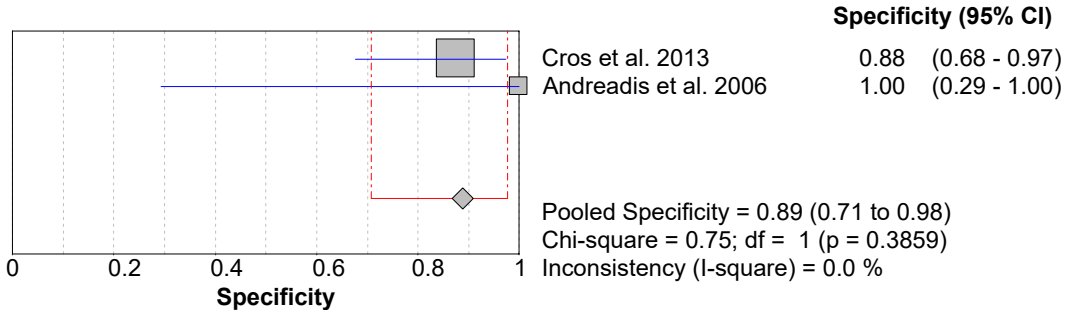

C

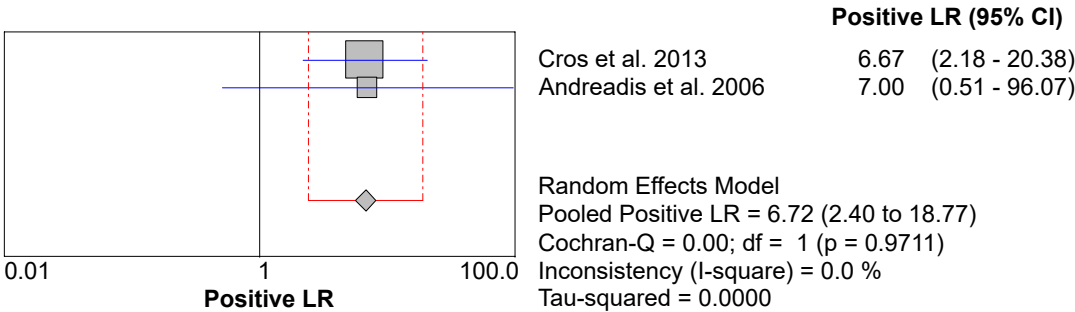

D

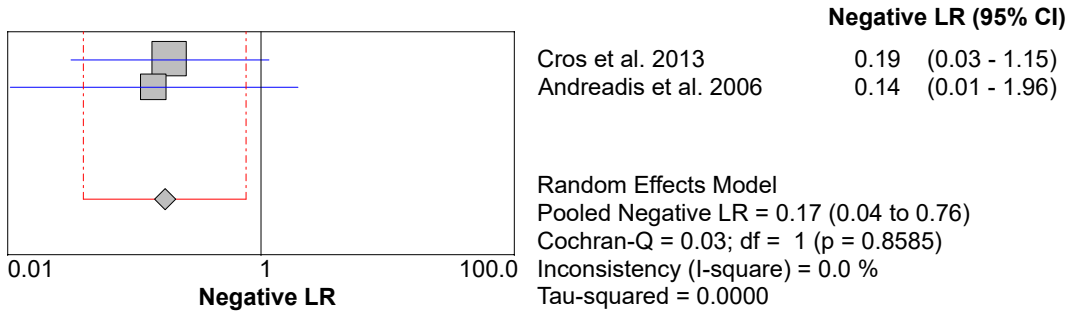

E

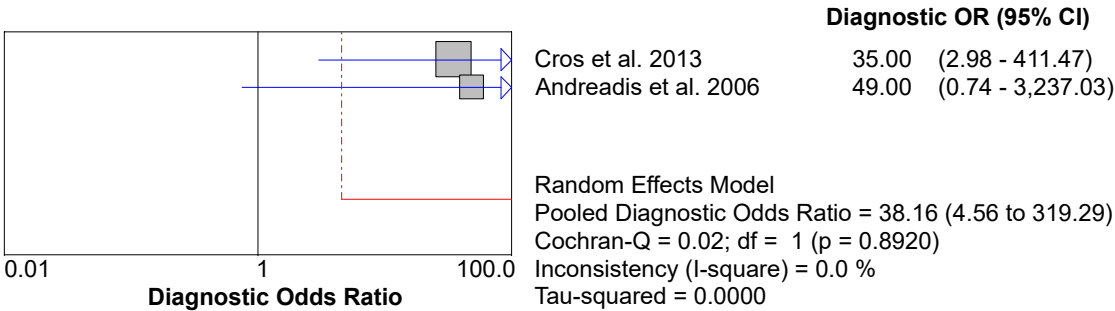

F

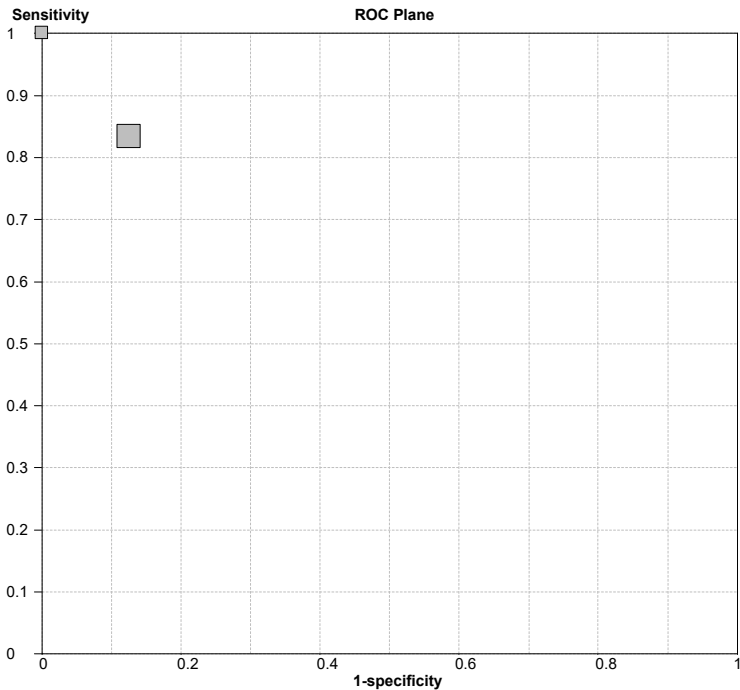

Supplement: Supplementary file 1 [file genes-16-00037-s001.zip › genes-3374333_Supplementary figures-Revised/Supplementary_Figure_9_KIT_EMC_MEC.pdf]
